# The Antiproliferative Activity of Tatridin A Against Prostate Cancer Cells Is Lost in Acid Medium by Transformation to Desacetyl-β-Cyclopyrethrosin

**DOI:** 10.3390/jox15050161

**Published:** 2025-10-09

**Authors:** Cecilia Villegas, Rebeca Pérez, Camilo Céspedes-Méndez, Viviana Burgos, Ricardo Baggio, Sebastián Suárez, Bernd Schmidt, Cristian Paz

**Affiliations:** 1 Department of Biological and Chemical Sciences, Faculty of Natural Resources, Catholic University of Temuco, Rudecindo Ortega 02950, Temuco 4780000, Chile; cecilia.villegas@uct.cl; 2 Pharmaceutical Chemistry Program, Faculty of Health Sciences, Autonomous University of Chile, Avenida Alemania 01090, Temuco 4780000, Chile; rebeca.perez@cloud.uautonoma.cl (R.P.); camilo.cespedes@uatonoma.cl (C.C.-M.); 3 Faculty of Health, School of Medical Technology, Santo Tomás University, Temuco 4780000, Chile; vburgos7@santotomas.cl; 4 Research and Applications Management, Constituyentes Atomic Center, National Atomic Energy Commission, San Martín, Buenos Aires B1650LWP, Argentina; baggio.ricardo@gmail.com; 5 Department of Analytical Chemistry and Instrumental Analysis, Faculty of Sciences, Francisco Tomás y Valiente St. No. 7, Campus of Excellence, Autonomous University of Madrid, 28049 Madrid, Spain; sebastian.suarez@uam.es; 6 Institute of Chemistry, University of Potsdam, Karl-Liebknecht-Straße 24-25, D-14476 Potsdam, Germany; bernd.schmidt@uni-potsdam.de; 7 Laboratory of Natural Products & Drug Discovery, Center CEBIM, Department of Basic Sciences, Faculty of Medicine, University of La Frontera, Temuco 4780000, Chile

**Keywords:** tatridin A, prostate cancer, NF-κB inhibition, sesquiterpene lactones, germacranolide, eudesmanolide

## Abstract

**Background:** Prostate cancer (PC) progression is strongly driven by dysregulated signaling pathways, with NF-κB playing a central role. Sesquiterpene lactones have been reported to modulate this pathway. This study evaluated and compared the cytotoxic effects of two structurally distinct sesquiterpene lactones: Tatridin A, a germacranolide, and desacetyl-β-cyclopyrethrosin, a eudesmanolide derivative. Their mechanisms of action were also examined, focusing on oxidative stress induction and NF-κB modulation. **Methods:** Chemical structures were confirmed by NMR and X-ray crystallography. Cytotoxicity was assessed in DU-145 and 22Rv1 PC cells using real-time cell analysis. Reactive oxygen species (ROS) and mitochondrial membrane potential (ΔΨm) were measured with fluorometric assays. NF-κB activity was determined in THP-1 reporter cells and by Western blot of IκBα phosphorylation. **Results:** Tatridin A markedly reduced viability, showing lower IC_50_ values (81.4 ± 2.7 µM in DU-145 and 50.7 ± 1.9 µM in 22Rv1 cells) than desacetyl-β-cyclopyrethrosin (166.9 ± 3.2 µM and 290.3 ± 8.3 µM, respectively). It also inhibited proliferation at markedly lower concentrations, with clonogenic IC_50_ values of 7.7 µM in DU-145 and 5.24 µM in 22Rv1cells. Both compounds increased ROS, but tatridin A induced earlier and stronger responses and ΔΨm loss. Furthermore, tatridin A more effectively inhibited NF-κB signaling than classical inhibitors. **Conclusions:** Tatridin A exerts cytotoxic effects through oxidative stress, mitochondrial impairment, and NF-κB inhibition, supporting the therapeutic potential of germacranolides for the treatment of advanced PC.

## 1. Introduction

Prostate cancer (PC) is one of the leading causes of oncologic mortality worldwide, with a projected incidence rising from 1.4 million cases in 2020 to 2.9 million in 2040, and a mortality that could reach 700,000 deaths annually [1]. Its development is closely related to androgen receptor (AR) activation and aberrant nuclear factor kappa B (NF-κB) signaling, which favors tumor progression, aggressiveness, and resistance to treatments [2,3]. Current therapies, based on AR inhibition and chemotherapy, present limitations due to acquired resistance and adverse effects [4], highlighting the need for new, more effective, and better tolerated therapeutic strategies.

NF-κB is actively involved in PC, playing a crucial role in tumor progression and disease relapse [5,6]. Its anomalous activation is partially associated with the cellular stress experienced by cancer cells. In this context, during conditions of moderate oxidative stress, reactive oxygen species (ROS) induce phosphorylation and degradation of IκB, leading to NF-κB activation [7]. Phosphorylation of Ser276 on RelA (p65) has been shown to be ROS-dependent, which is essential for transcription of NF-κB target genes [8]. However, more severe oxidative stress can reduce NF-κB activity, since an excess of ROS interferes with the activation of IκBα kinases (IKKs) that regulate the pathway [9]. NF-κB activation has been linked to castration resistance in advanced stages of PC [10,11], with elevated levels of nuclear p65, a marker of its activation, being observed in comparison with normal prostate tissue [6,12]. Given its central role in the pathogenesis of PC, NF-κB presents itself as a key therapeutic target, with potential for the development of new strategies based on natural molecules that regulate this signaling and favor tumor cell apoptosis, particularly in advanced PC [13,14,15].

The germacranolide sesquiterpene lactones, present in the *Asteraceae* family, possess a ten-membered carbocyclic nucleus fused to an unsaturated lactone. Their biological activity is due to the α-methylene-γ-lactone, which reacts with the nucleophilic sites of proteins by Michael addition, and to the presence of polar groups that modulate their interaction [16,17]. Some germacranes induce apoptosis in cancer cells by altering the redox balance and activating the mitochondrial pathway, as well as inhibiting NF-κB and signal transducer and activator of transcription 3 (STAT3), making them promising anti-cancer therapeutic tools [18,19]. In contrast, eudesmanolide-type sesquiterpene lactones are derived from germacranes by intramolecular cyclization that confers their structure with increased rigidity. This structural difference may significantly influence their interaction with key proteins in inflammatory signaling, by modifying their affinity for nucleophilic residues such as cysteine thiol groups [20,21].

In this study tatridin A, a germacranolide sesquiterpene lactone, was isolated from *Podanthus mitiqui*, a medicinal plant endemic to Chile [22,23], while the eudesmanolide-type sesquiterpene lactone desacetyl-β-cyclopyrethrosin (**2**) resulted from a cyclization reaction of tatridin A, catalyzed by traces of HCl formed through partial decomposition of the solvent chloroform. Tatridin A exhibits cytotoxic effect in cell lines such as HL-60 and U937, inducing apoptosis by DNA fragmentation with low genotoxicity, suggesting an important therapeutic potential [24]. In addition, its ability to inhibit phosphoglycerate kinase 1 (PGK1) alters tumor metabolism, potentially increasing the susceptibility of cancer cells to chemotherapy or cell death [25]. However, its effect on advanced PC remains unexplored. This research aimed to assess the cytotoxic effects of tatridin A and to compare its efficacy with desacetyl-β-cyclopyrethrosin in DU-145 and 22Rv1 prostate cancer cell lines. In addition, we sought to elucidate the underlying mechanisms, focusing on ROS production, mitochondrial impairment, and the modulation of NF-κB, a critical signaling pathway implicated in prostate cancer cell survival and tumor progression.

## 2. Materials and Methods

### 2.1. Isolation and Structure Elucidation of Tatridin A and Desacetyl-β-Cyclopyrethrosin

Tatridin A was isolated from the aerial parts of *Podanthus mitiqui* collected in Concepcion, VIII Region of Chile, as described previously [22,23]. Desacetyl-β-cyclopyrethrosin) was obtained from compound **1** by its acid-catalyzed rearrangement in CDCl_3_ (Merck, Darmstadt, Germany) during NMR analysis, crystals of compound **2** were subsequently isolated after recrystallization from EtOAc (Merck, Darmstadt, Germany). Pure compound **2** was then used for all subsequent biological assays. Structure elucidation of tatridin A and desacetyl-β-cyclopyrethrosin was accomplished by 1D and 2D-NMR spectroscopy in deuterated acetone (Sigma-Aldrich, St. Louis, MO, USA). All NMR spectra were recorded at 500 MHz (^1^H NMR spectroscopy) and 125 MHz (^13^C NMR spectroscopy), respectively, using a Bruker Avance NEO 500 spectrometer (BrukerBiospin GmbH, Rheinstetten, Germany). Signal assignments are based on the 2D-NMR experiments H,H-COSY, NOESY, HSQC, and HMBC. Copies of all spectra, full signal assignments, and comparison with reference data available in the literature are provided in the Supporting Information. Copies of NMR spectra for tatridin A are provided in Figures S3–S11 and for desacetyl-β-cyclopyrethrosin in Figures S12–S17.

### 2.2. X-Ray Single-Crystal Structure

Suitable single crystals were mounted over a mylar loop (MiTeGen, Ithaca, NY, USA) using paratone oil (Hampton Research, Aliso Viejo, CA, USA) and data were collected at room temperature using a Gemini A diffractometer (Oxford Diffraction, Abingdon, UK), equipped with Eos charge-coupled device (CCD) detector with graphite-monochromated Cu Kα (λ = 1.54184 Å) radiation, available at the Institute of Chemistry, Physics of Materials, Environment and Energy (INQUIMAE), Faculty of Exact and Natural Sciences, University of Buenos Aires, Argentina (FCEN-UBA). CrysAlisPro software 1.171.41.112 (Oxford Diffraction), was used to collect initial frames for the determination of the unit cell, and subsequently, the program was used to plan data collection [26]. After collection, data reduction was carried out in the CrysAlisPro suite, followed by absorption correction. See Supporting Information (SI) for specific refinement details of each structure. Complete crystallographic data sets have been deposited in Crystallographic Information File (CIF) format at the Cambridge Structural Database (CSD), as deposition numbers 2,423,080 (Structure II) and 2,423,081 (Structure I) [27]. Geometrical calculations and molecular representations, images and tables were performed by and generated with the software programs MERCURY 4.3.1 [28] and PLATON [29].

### 2.3. Cell Culture

Human prostate DU-145 and 22Rv1 epithelial cells were obtained from the American Type Culture Collection (ATCC) and cultured in RPMI-1640 medium (Cytiva, Marlborough, MA, USA; SH30027.01) supplemented with 10% fetal bovine serum (FBS) (Cytiva; SH30071.03), 100 U/mL penicillin (Sigma-Aldrich, St. Louis, MO, USA), and 0.1 mg/mL streptomycin (Cytiva; SV30010) at 37 °C in a humidified atmosphere with 5% CO_2_. DU-145 cells are considered part of the gold standard triad of PC cell culture lines because they represent a model of advanced androgen-independent PC model with active NF-κB signaling [30,31]. In contrast, the 22Rv1 cell line are characterized by AR and prostate-specific antigen (PSA) expressions, despite their ability to proliferate independently of androgen [32].

### 2.4. Real-Time Cell Death Assay

Real-time cytotoxicity of tatridin A and desacetyl-β-cyclopyrethrosin were systematically assessed. DU-145 and 22Rv1 cell lines (20,000 cells per well) were seeded into 96-well plates and exposed to increasing concentrations of tatridin A and desacetyl-β-cyclopyrethrosin (6–200 μM) in RPMI-1640 medium supplemented with 10% FBS and 0.1% (*v*/*v*) DMSO (Sigma-Aldrich, St. Louis, MO, USA; D8418). The experiments were executed utilizing the IncuCyte^®^ S3 live-cell analysis system (Bohemia, NY, USA), employing 30 nM Sytox Green dye (Invitrogen, Carlsbad, CA, USA, S7020) as a marker for cellular mortality [33,34]. The assessment of dead cells was executed automatically every hour for a total duration of 48 h based on the green fluorescence and IC_50_ values were calculated using GraphPad Prism 8.0 (GraphPad Software, LLC, San Diego, CA, USA). The confluence area of the cellular monolayer, measured as the percentage of the image area covered by cells, was determined using IncuCyte^®^ v2019B software provided by the Advanced Microscopy Center (CMA) BioBio at the University of Concepción. Chile. Cell size analysis was performed using the mean object area (µm^2^) data obtained with the IncuCyte^®^ system. For each cell line (22Rv1 and DU-145), compound, and concentration, independent measurements from two biological replicates were considered after 48 h of exposure to the compounds. Mean values and standard deviations were calculated. Comparisons with the untreated control were assessed using one-way analysis of variance (ANOVA) followed by Dunnett’s post hoc test, with differences considered significant at *p* < 0.05.

To integrate both proliferation and cytotoxicity into a single readout, we computed net viability from the IncuCyte^®^ dataset by combining confluence (cell-covered area, as a measure of proliferation) and Sytox Green-positive cells (as a measure of cell death) acquired from the same wells. Net viability was calculated according to the following formula:

$$Net\;viability\;\left(\%\right)\;=\;\frac{Confluence\;(treated)\;\times \;(1-Sytox\;positive)}{Confluence\;control\;(not\;treated)}\times 100$$

### 2.5. Selection of the Standard Working Concentration

The working concentration for tatridin A and desacetyl-β-cyclopyrethrosin was set at 50 μM for the 24 h experiments. At this concentration, tatridin A caused approximately 50% cell death, while desacetyl-β-cyclopyrethrosin showed limited cytotoxicity. Using the same concentration for both compounds allowed for a direct comparison of their biological effects under standardized conditions.

### 2.6. Clonogenic Assays

The antiproliferative activity of compounds tatridin A and desacetyl-β-cyclopyrethrosin at sub-IC_50_ concentrations was assessed using a clonogenic assay. Cells were seeded at a density of 1000 cells per well in 12-well plates and exposed to concentrations selected as “sub-toxic”, (ranging from 1.56 to 25 µM) in RPMI-1640 medium containing 10% FBS, 100 U/mL penicillin, and 0.1 mg/mL streptomycin meaning doses that do not acutely compromise short-term viability but still allow long-term assessment of proliferative capacity. After a 3 h incubation, the compounds were removed and cells were maintained in fresh RPMI-1640 medium for 11 d, with the medium replaced every 4 days. Colonies were then fixed with 80% ethanol (Merck, Darmstadt, Germany) for 15 min at room temperature, stained with 0.5% crystal violet (Sigma-Aldrich, St. Louis, MO, USA) for 20 min, and subsequently photographed using a standard digital camera [35]. The total colony area for each condition was quantified using the Image J software V.1.49 (NIH, Bethesda, MD, USA) [36].

### 2.7. Reactive Oxygen Species Measurement

Cells were seeded at a density of 15,000 cells per well in a black flat-bottomed 96-well plate and maintained at 37 °C with 5% CO_2_ in RPMI-1640 medium without phenol red (Gibco, Thermo Fisher Scientific, Waltham, MA, USA; 11835030) containing 10% FBS, 100 U/mL penicillin, and 0.1 mg/mL streptomycin. After 24 h, the cells were incubated with 5 μM of 2′,7′-dichlorodihydrofluorescein diacetate (H_2_DCFDA) (Tocris Bioscience, Bristol, UK; 5935) for 30 min, following the manufacturer’s instructions and the protocol described by Ajayi et al. [37]. Subsequently, cells were treated with 50 μM tatridin A and desacetyl-β-cyclopyrethrosin, using 0.1% DMSO as the control. Fluorescence was measured hourly for 20 h with the VICTOR^®^ Nivo™ microplate reader (PerkinElmer, Waltham, MA, USA) at 495 nm excitation and 520 nm emission.

### 2.8. Mitochondrial Membrane Potential Analysis

This analysis was performed in real-time using the IncuCyte^®^ analysis system with the Image-iT^®^ tetramethylrhodamine methyl ester reagent (TMRM; Invitrogen, Carlsbad, CA, USA; I34361) to monitor changes in mitochondrial membrane potential (ΔΨm) based on relative fluorescence intensity. The procedure followed the manufacturer’s guidelines, with modifications based on the protocol outlined by Creed et al. [38]. DU-145 and 22Rv1 cells were seeded at 20,000 cells per well in 96-well plates and incubated for 24 h. They were stained with 100 nM TMRM and incubated for 30 min. Treatments with 50 μM tatridin A, desacetyl-β-cyclopyrethrosin, or 0.1% DMSO (control) were applied. Fluorescence intensity was recorded every hour for 24 h using the IncuCyte^®^ software (v.2019B).

### 2.9. Analysis of NF-κB Activation

NF-κB Activation was evaluated using an alkaline phosphatase reporter assay in human monocytic THP1-Blue™ cells (InvivoGen, San Diego, CA, USA), stably modified to express a secreted embryonic alkaline phosphatase (SEAP) gene under the control of an NF-κB-inducible promoter. THP1-Blue cells were seeded at 100,000 cells per well in a 96-well plate and pretreated with tatridin A, desacetyl-β-cyclopyrethrosin, or 0.1% DMSO (vehicle control) for 30 min. This was followed by stimulation with 100 ng/mL lipopolysaccharide (LPS) (InvivoGen, San Diego, CA, USA); for 24 h, as described by Cantini et al. [39]. Alkaline phosphatase activity was quantified from cell supernatants using the QUANTI-Blue reagent (InvivoGen, San Diego, CA, USA), following the manufacturer’s instructions. Absorbance was measured at 655 nm and compared to LPS-treated positive controls. The percentage of NF-κB pathway inhibition was subsequently calculated relative to the LPS-only condition, which was set as 100% activation.

### 2.10. Western Blot Analysis

DU-145 cells (1 × 10^6^ cells per well) were seeded and treated with 50 µM of tatridin A, desacetyl-β-cyclopyrethrosin, with and without LPS (1 µg/mL) and BAY 11-7082 (Sigma-Aldrich, St. Louis, MO, USA). This compound is a phenyl vinyl sulfone derivative whose structure is well described in the literature [40]. BAY 11-7082 irreversibly inhibits TNF-α-induced IκB-α phosphorylation, thereby blocking NF-κB activation [41]. Following an incubation period of 1 h, the cells were rinsed twice with cold Phosphate-buffered saline (PBS; Gibco, Thermo Fisher Scientific, Waltham, MA, USA). Subsequently, they were lysed using the RIPA Lysis Buffer System^®^ (Santa Cruz Biotechnology, Inc., Dallas, TX, USA; sc-24948), which was supplemented with the protease and phosphatase inhibitor cocktails provided in the RIPA Lysis Buffer System^®^. The cell lysates were collected by scraping and transferred to 1.5 mL tubes, followed by centrifugation at 12,000× *g* for 20 min. The supernatant was carefully removed, and protein concentration was determined using the Pierce™ BCA protein assay kit (Thermo Fisher Scientific, Waltham, MA, USA). Next, 30 μg of protein samples were subjected to electrophoresis and transferred to a polyvinylidene fluoride (PVDF) membrane (Millipore, Burlington, MA, USA). The membrane was incubated overnight at 4 °C with the primary antibodies in Tris-buffered saline (TBS; Thermo Fisher Scientific, Waltham, MA, USA) containing Tween 20^®^ (Sigma-Aldrich, St. Louis, MO, USA). The following antibodies were used: anti-IκBα (1:1000, Cell Signaling Technology, Danvers, MA, USA; #4812), anti-phospho-IκBα (P-IκBα) (1:1000, Cell Signaling Technology; #9246), and anti-α-tubulin (1:1000, Santa Cruz Biotechnology Inc, Dallas, TX, USA; sc-5286). Immunoreactive bands were detected using secondary antibodies conjugated with horseradish peroxidase (anti-mouse IgG, 715-035-150; anti-rabbit IgG, 711-035-152, both 1:10,000, Jackson ImmunoResearch Inc., West Grove, PA, USA) and visualized through chemiluminescence using the SuperSignal™ West Pico PLUS chemiluminescent substrate (Thermo Fisher Scientific, Waltham, MA, USA). Images were obtained with a G:Box chemi XRQ gel doc system (Syngene, Frederick, MD, USA) and band densitometry analysis was performed with Image J software V.1.49 (NIH).

## 3. Results

### 3.1. Structure Elucidation of Tatridin A and Desacetyl-β-Cyclopyrethrosin

**Figure 1:**
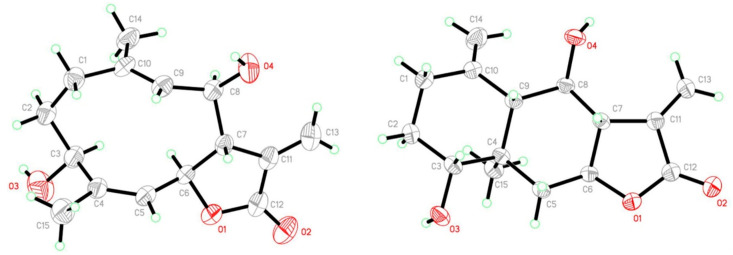
Molecular structure of tatridin A (**left**) and desacetyl-β-cyclopyrethrosin (**right**). Displacement ellipsoids are shown at the 40% probability level.

Tatridin A is a germacranolide sesquiterpene lactone that was first isolated from *Artemisia arbuscula* ssp. [42], *Artemisia tridentata* [43], and *Tanacetum vulgare* [44]. As reported earlier in the literature, tatridin A shows activity against various pathogenic bacteria, including *Staphyllococcus aureus*, and fungi at low micromolar concentrations [45]. It also inhibits the growth of HL-60 and U937 human myeloid leukemia cells at the micromolar level [24]. High-resolution ^1^H- and ^13^C-NMR data and a full signal assignment were also reported [46], but no copies of NMR spectra and primary data have so far have been published. Tatridin A was identified in this work by analysis of its 1D- and 2D-NMR spectra (Table 1 and Supporting Information), and this identification was unambiguously corroborated by single-crystal X-ray structure determination performed on suitable crystals obtained using EtOAc (Merck, Darmstadt, Germany) as the recrystallization solvent. An Oak Ridge Thermal Ellipsoid Plot (ORTEP) type view of the molecule, displaying 40% probability ellipsoids, is shown in Figure 1 (left). Table S1 presents relevant crystal and refinement data; bond distances and angles are unexceptional. The molecular structure of tatridin A had earlier been determined by single-crystal X-ray structure analysis [47].

**Scheme 1:**
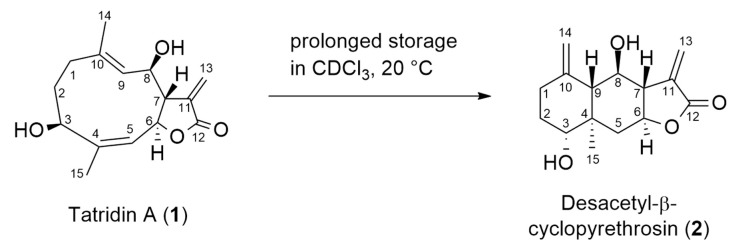
Formation of desacetyl-β-cyclopyrethrosin (**2**) from tatridin A (**1**) after prolonged storage in CDCl_3_ at 20 °C (approximately 3 months).

When the NMR spectra of tatridin A were recorded in deuterated acetone, we noticed that a second set of signals appeared (Figure S12). The intensity of these signals increased over time, whereas the signals originating from tatridin A decreased in intensity, until eventually only the signals from the new product were observed. Based on its 1D- and 2D-NMR spectra, compound **2** was identified as desacetyl-β-cyclopyrethrosin (Scheme 1). This structural assignment was unambiguously confirmed by single-crystal X-ray analysis Figure 1 (right), performed on single crystals isolated from the NMR sample and obtained after recrystallization from EtOAc. Compound **2** crystallizes (without solvent molecules) in the orthorhombic space group P2_1_2_1_2_1_. The configuration of the chiral centers of compound 2 was unambiguously determined in this study, revealing a slight discrepancy with the first report of this compound [48] (Figure S1). Desacetyl-β-cyclopyrethrosin had earlier been reported as a natural product, isolated, e.g., from *Mikania* sp. [49], *Brocchia cinerea* [50], *Anthemis altissima* [51], or very recently from *Staehelina uniflosculosa* [52] and *Inula britannica* [53]. Antibacterial activity as well as some cytotoxic effect potential have been reported for desacetyl-b-cyclopyrethrosin (**2**) [45].

### 3.2. Mechanistic Proposal for the Isomerization of Tatridin A to Desacetyl-β-Cyclopyrethrosin

Chloroform is prone to slow decomposition in the presence of oxygen and light. The products of this oxidation are phosgene and HCl [54]. The same decomposition occurs also with CDCl_3_, the most common of all solvents for NMR spectroscopy that had originally also been used by us for recording the NMR spectra of tatridin A. Acid impurities in CDCl_3_ can affect the NMR spectra of compounds with acid-labile functional groups inducing acid-catalyzed transformations [55], and it can be assumed that this is the case here. Indeed, there is precedence in the literature for acid-mediated cyclization reactions of germacranolides to eudesmanolides: For instance, the eudesmanolide sesquiterpene desacetyl-β-cyclopyrethrosin had been synthesized from the germacranolide pyrethrosin by treatment with *p*-toluene sulfonic acid through a cyclization-elimination mechanism [56]. Jain and McCloskey reported that the germacranolide costunolide undergoes cyclization to two isomeric cyclocostunolides in the presence of a cationic ion exchange resin [57,58]. Barrero et al. found that a germacranolide isolated from *Tanacetum annum* reacts to two eudesmanolides in the presence of thionyl chloride, one with a C-C double bond in *exo*-, and one in *endo*-position [59]. In addition to these examples, some other reports describe transannular cyclizations of germacranolides, mediated either by Brønsted- or by Lewis-acids that have in some cases been used as synthesis steps on a preparative scale [60,61,62,63,64,65,66,67].

**Scheme 2:**
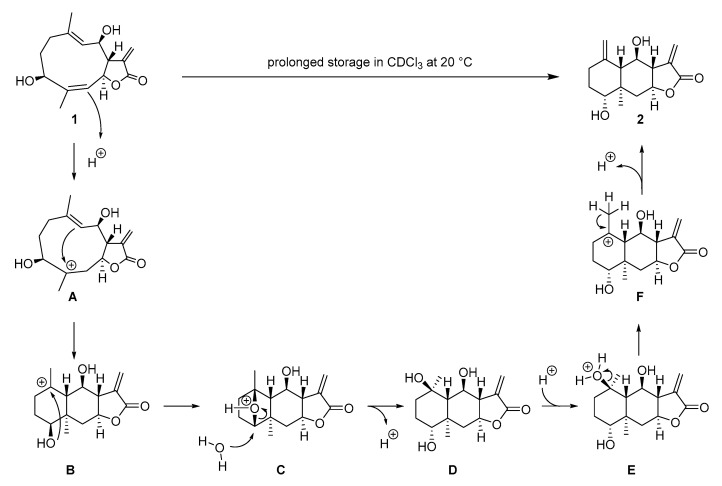
Mechanistic proposal for the acid-catalyzed rearrangement of tatridin A (**1**) into desacetyl-β-cyclopyrethrosin (**2**) after prolonged storage in CDCl_3_ at 20 °C (approximately 3 months). (**A**) stabilized tertiary carbocation after protonation of C4=C5; (**B**) carbocation from transannular cyclization (C4–C9); (**C**) protonated bicyclic oxonium from intramolecular attack of C3–OH; (**D**) tertiary alcohol at C10 after SN2-type water attack; (**E**) oxonium ion from protonation of D; (**F**) tertiary carbocation after E1-elimination of water.

A plausible mechanistic explanation for the rearrangement of tatridin A to desacetyl-b-cyclopyrethrosin must account for both the transannular cyclization between C4 and C9 and the inversion of the configuration at C3 (Scheme 2). We propose a protonation of the C4=C5 double bond in the first step to furnish the stabilized tertiary carbenium ion A. Transannular cyclization leads to another tertiary carbenium ion B. The following steps account for the inversion of configuration at C3. Intramolecular attack of the C3–OH group yields the strained and protonated bicyclic system C, which reacts by nucleophilic S_N2_-type attack of water at C3, to give a tertiary alcohol at C10 (intermediate D). Protonation of the tertiary alcohol D to oxonium ion E, and subsequent E_1_-elimination of water gives the tertiary carbenium ion F, which eventually reacts to desacetyl-b-cyclopyrethrosin by cleavage of a proton in b-position. The final steps of this mechanistic proposal are supported by a report by Cardona et al., who found that eudesmanolide D, resulting from an acid-catalyzed transannular cyclization of the germacrane epoxide pyrethrosin, reacts further in the presence of acid to desacetyl-b-cyclopyrethrosin [56].

### 3.3. Tatridin A Shows Cytotoxic Effects Against DU-145 and 22Rv1 Cell Lines

**Figure 2:**
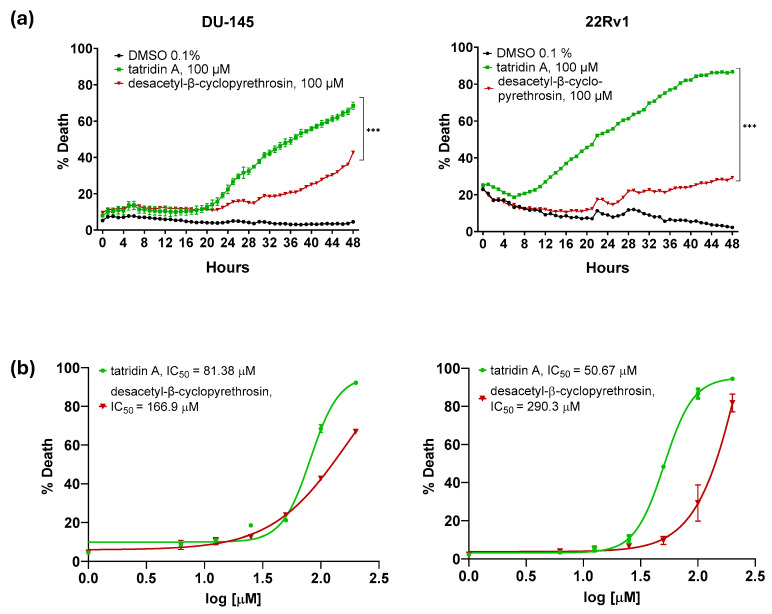
Cytotoxic effects against DU-145 and 22Rv1 cells. (**a**) Real-time plots for cell death measurements (48 h, %) in cells exposed to 100 μM tatridin A (green line) and desacetyl-β-cyclopyrethrosin (red line). Data are presented as mean ± standard deviation (SD) (*n* = 3). Statistical analysis was performed using two-way ANOVA followed by Tukey’s post hoc test. *** *p* < 0.0001 for tatridin A vs. desacetyl-β-cyclopyrethrosin at 48 h. (**b**) Dose–response curves evaluated using IncuCyte^®^ for 48 h. The percentage of cell death is plotted as a function of the logarithmic concentration of the compounds, with the IC_50_ value for each compound indicated on the graph.

Real-time cytotoxicity assessment using the IncuCyte^®^ live-cell analysis system, as indicated that tatridin A exhibits cytotoxic effect against advanced PC cell lines compared to desacetyl-β-cyclopyrethrosin and DMSO (*p* < 0.0001; Figure 2a). In DU-145 cells, exposure for tatridin A promoted a progressive elevation of cell death, reaching approximately 60% at 48 h. In contrast, desacetyl-β-cyclopyrethrosin showed a less pronounced effect, with cell death approaching 30% in the same period. Similarly, in 22Rv1 cells, tatridin A induced higher cell death (approximately 80%) compared with desacetyl-β-cyclopyrethrosin (approximately 30%), suggesting a higher cytotoxicity of the former. In addition, 22Rv1 cells were more sensitive, showing an earlier onset of cell death compared with DU-145 cells. The dose–response curve plots displayed in Figure 2b reinforce these findings. In DU-145 cells, tatridin A presented an IC_50_ of 81.38 ± 2.7 µM, while desacetyl-β-cyclopyrethrosin showed an IC_50_ of 166.9 ± 3.2 µM, indicating a lower cytotoxic effect. In 22Rv1 cells, the IC_50_ of tatridin A was 50.67 ± 1.9 µM, significantly lower than that of desacetyl-β-cyclopyrethrosin, 290.3 ± 8.3 µM, suggesting a higher sensitivity of this cell line for tatridin A.

### 3.4. Tatridin A Reduces the Proliferative Activity of DU-145 Cells and 22Rv1 Cells

**Figure 3:**
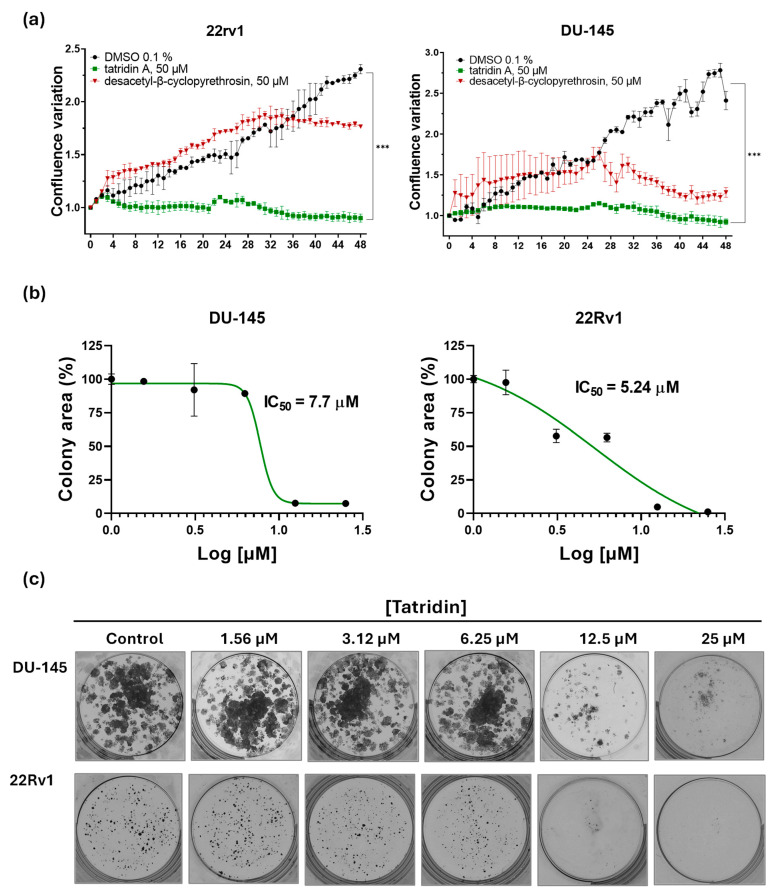
Antiproliferative activity of DU-145 and 22Rv1 cells. (**a**) Time-course analysis of cell confluence in the DU-145 and 22Rv1 cell lines treated with tatridin A and desacetyl-β-cyclopyrethrosin at 50 μM. Data are presented as mean ± SD (*n* = 3). Statistical analysis was performed using two-way ANOVA followed by Tukey’s post hoc test at 48 h. *** *p* < 0.0001 for tatridin A vs. DMSO control at 48 h. (**b**) IC_50_ values for colony formation were determined for tatridin A. The percentage of colony area was quantified using the ImageJ software V.1.49, enabling precise analysis of treatment effects on long-term cell viability and proliferation. (**c**) Representative images of colonies stained with crystal violet after 11 d of culture of the DU-145 and the 22Rv1 cell lines exposed to following tatridin A exposure at non-cytotoxic effect concentrations (1.56–25 µM) for 3 h.

The real-time confluence measurements by IncuCyte^®^ showed that tatridin A (50 µM) significantly suppressed proliferation in both DU-145 and 22Rv1 cells, maintaining confluence close to baseline throughout the 48 h period when compared with the DMSO control (*p* < 0.0001; Figure 3a). In contrast, desacetyl-β-cyclopyrethrosin displayed only partial inhibitory effects. in DU-145, it slowed proliferation after an initial increase, stabilizing at values well below the control, whereas in 22Rv1 cells it allowed continued growth, before reaching a plateau. These results confirm that tatridin A exerts a markedly stronger antiproliferative effect than desacetyl-β-cyclopyrethrosin. Germacranolides have been described to exhibit cytostatic and proapoptotic effects, including induction of cell cycle arrest in PC cells [68].

Analysis of mean object area (µm^2^) from IncuCyte revealed a significant, dose-dependent reduction in cell size for both compounds in 22Rv1 and DU-145 cells, but only at the highest concentrations tested. In 22Rv1, tatridin A (ANOVA, Fisher statistic F = 26.37, *p* = 0.00018) and desacetyl-β-cyclopyrethrosin (F = 55.86, *p* = 1.47 × 10^−5^) markedly reduce cell size. Similar effects were observed in DU-145 cells, where tatridin A (F = 67.41, *p* = 0.000008) and desacetyl-β-cyclopyrethrosin (F = 15.61, *p* = 0.00098) significantly reduced cell size. No significant size changes were observed at lower concentrations, consistent with the clonogenic assay design.

To provide a more comprehensive assessment of the impact of the compounds on cell count, we supplemented this evaluation with an integrated net viability analysis using our IncuCyte^®^ dataset. This approach combined proliferation (cell confluence) and cytotoxicity (Sytox Green-positive) in the same wells, normalized to untreated controls. The resulting dose–response curves confirmed the trends, showing a progressive decrease in net viability with increasing concentrations. The net-viability IC_50_ values were 53.66 ± 1.8 μM (DU-145 cells) and 35.38 ± 2.1 μM (22Rv1 cells) for tatridin A, and 101.20 ± 2.9 μM (DU-145 cells) and 80.98 ± 3.7 μM (22Rv1 cells) for desacetyl-β-cyclopyrethrosin (Table 2).

Since tatridin A inhibited cell proliferation at 48 h, we investigated whether prolonged exposure would prevent colony formation in advanced PC cells. The colony formation curves (Figure 3b) confirmed the antiproliferative effect of tatridin A, as it effectively inhibited colony formation in the DU-145 and the 22Rv1 cell lines. The IC_50_ values were 7.7 ± 1.4 μM in DU-145 cells and 5.24 ± 0.95 μM in 22Rv1 cells, indicating that the 22Rv1 cell line is more sensitive to this compound. Such effect was illustrated by representative images of colonies stained with crystal violet (Figure 3c). After 11 d of culture, a concentration-dependent reduction in colony formation was observed in both cell lines. In DU-145 cells, concentrations starting at 6.25 µM significantly reduced colony number and size. In the 22Rv1 cells, even the lowest concentrations of tatridin (3.12 µM) showed an inhibitory effect, with a drastic colony formation decrease observed from 12.5 µM.

### 3.5. Tatridin A Treatment Increase Cellular Reactive Oxygen Species Production and Decreases Mitochondrial Membrane Potential

**Figure 4:**
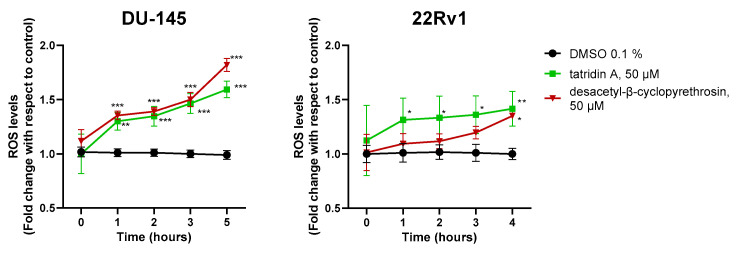
Effects of tatridin A and desacetyl-β-cyclopyrethrosin on cellular ROS production in DU-145 and the 22Rv1 cell lines. Quantification of ROS production using the fluorescent probe H_2_DCFDA. * *p* < 0.05, ** *p* < 0.01, *** *p* < 0.001.

**Figure 5:**
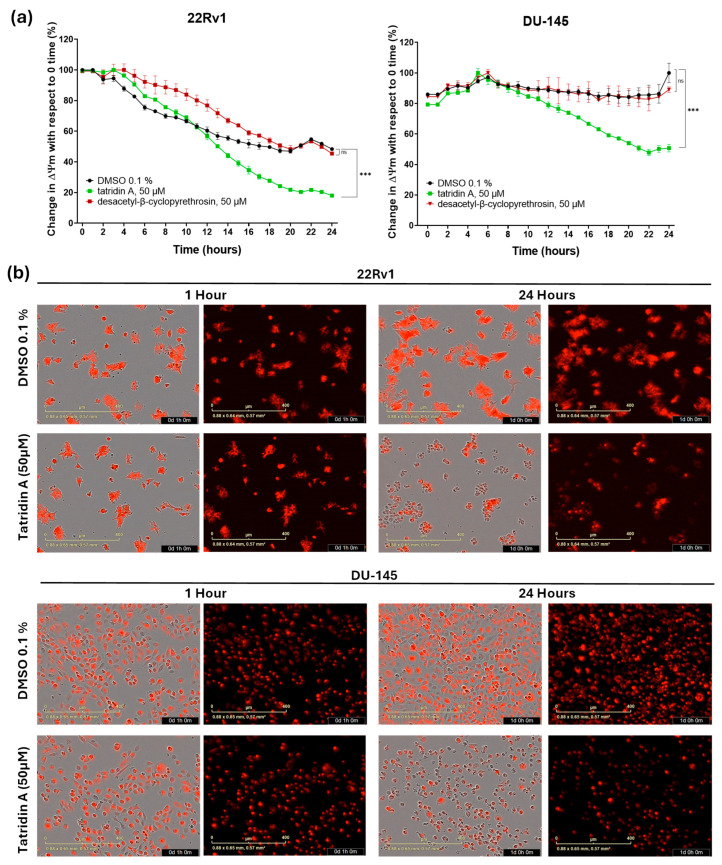
Effects of tatridin A and desacetyl-β-cyclopyrethrosin on ΔΨm in DU-145 and 22Rv1 cells. (**a**) Time-dependent reduction in ΔΨm after treatment with 50 μM compounds, measured by TMRM fluorescence relative to DMSO (0.1%). Data are mean ± SD (*n* = 3). Two-way ANOVA with Tukey’s post hoc test showed that tatridin A significantly decreased ΔΨm from 8 h in DU-145 and 11 h in 22Rv1 (*** *p* < 0.0001), this effect was not significant compared to its counterpart (ns). (**b**) Representative TMRM (red) and brightfield images at 1 h and 24 h confirm that fluorescence loss reflects ΔΨm dissipation rather than cell absence.

The results showed that tatridin A and desacetyl-β-cyclopyrethrosin significantly increased cellular ROS generation. Specifically, in DU-145 cells tatridin A and desacetyl-β-cyclopyrethrosin showed a progressive increase in ROS generation throughout the incubation period, with desacetyl-β-cyclopyrethrosin having the greatest effect on ROS generation at 5 h. In 22Rv1 cells, tatridin A showed a more potent effect during the first 3 h, while desacetyl-β-cyclopyrethrosin also significantly increased ROS levels production, although this effect was observed at a later time point (Figure 4). Regarding the impact of germacranolides on mitochondrial function, kinetic studies of depolarization revealed that tatridin A caused a significant decrease in ΔΨm in DU-145 and 22Rv1 cell lines compared to control, starting at 8 and 11 h of incubation, respectively (Figure 5a, plot). These findings were further corroborated by microscopic images acquired at the baseline (time 0) and 24 h, which showed a marked decrease in red fluorescence, indicative of ΔΨm loss (Figure 5b).

### 3.6. Tatridin A Reduces the Activity of the NF-κB Pathway More Efficiently than Eudesmane in the THP-1 Reporter Cell Line

**Figure 6:**
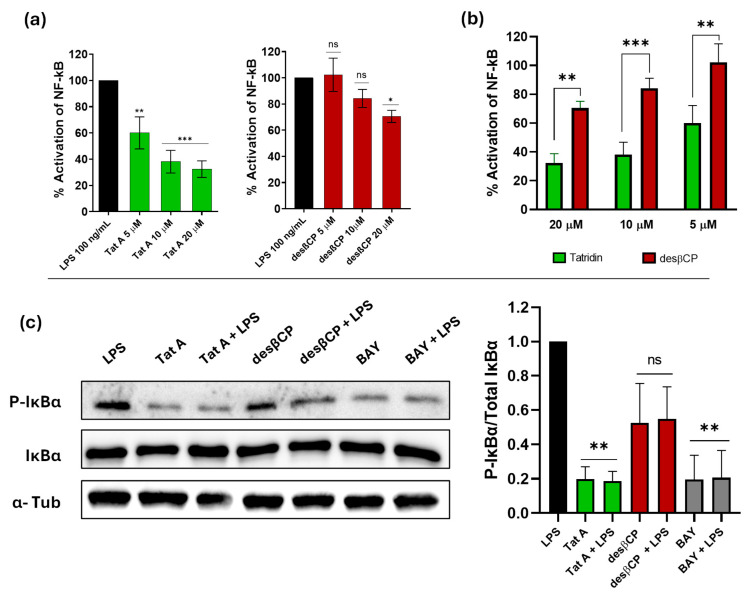
Effect of tatridin A (Tat A) and desacetyl-β-cyclopyrethrosin (desβCP) on the NF-κB pathway. (**a**) NF-κB responses were measured in THP1-Blue™ cells. NF-κB activation was expressed as the percentage of SEAP activity relative to the positive control (cells stimulated with LPS alone). Cells were incubated with LPS for 24 h. After incubation, NF-κB-induced SEAP activity was assessed. Data are shown as percentage based on OD at 650 nm (mean ± SD). (**b**) Comparison of the reduction in NF-κB activity upon treatment with tatridin A and desacetyl-β-cyclopyrethrosin at different concentrations (20, 10, and 5 μM). * *p* < 0.05, ** *p* < 0.01, *** *p* < 0.001. (**c**) Western blot analysis of IκBα and phospho-IκBα in DU-145 cells treated with tatridin A, desacetyl-β-cyclopyrethrosin, and BAY 11-7082 (BAY) in presence or absence of LPS for 1 h. The graph (right) shows the ratio of phospho-IκBα to IκBα. α-Tubulin (α-Tub) was used as a loading control. Significant differences between treatments and control (only LPS) are marked with asterisks (** *p* < 0.01), ns, not significant. All treatments were at 50 μM.

Inhibition of the NF-κB signaling pathway was evaluated in THP1-Blue™ reporter cells exposed to tatridin A and desacetyl-β-cyclopyrethrosin at 5, 10, and 20 μM, using SEAP production as a marker of activity. Stimulation with LPS activated the NF-κB pathway, reaching 100% SEAP production. In the case of tatridin A, SEAP production showed a significant decrease starting at 5 µM, reaching a reduction of approximately 40% (*p* < 0.01), while at 20 µM, activation was reduced to 70% (*p* = 0.001), indicating a dose-dependent inhibition (Figure 6a). In the case of desacetyl-β-cyclopyrethrosin, only a slight significant decrease was observed at 20 µM, reaching a reduction of approximately 26% (*p* < 0.05). Comparison between tatridin A and desacetyl-β-cyclopyrethrosin shows that tatridin A induces a much more marked inhibition of NF-κB activation compared to desacetyl-β-cyclopyrethrosin at concentrations starting at 5 µM (Figure 6b).

### 3.7. Tatridin A Inhibits IκBα Phosphorylation Akin to Other Classical NF-κB Inhibitors

In this study, inhibition of phosphorylated IκBα (P-IκBα) in DU-145 cells was assessed by Western blot analysis. Phosphorylation of IκBα served as a marker of NF-κB pathway activation. Stimulation with LPS increased IκBα phosphorylation, while treatment with tatridin A (50 µM) significantly reduced P-IκBα levels compared to the LPS control. Notably, its suppressive effect on NF-κB signaling was comparable to that of BAY 11-7082, a well-established NF-κB inhibitor (Figure 6c). This suggests that tatridin A inhibits the NF-κB pathway by preventing IκBα phosphorylation, possibly stabilizing non-phosphorylated IκBα and hindering the release of the p50/p65 dimer in DU-145 cells. In contrast, desacetyl-β-cyclopyrethrosin showed more limited inhibition and no significant differences, which could be related to structural differences and reduced binding affinity to key proteins in this inflammatory pathway.

## 4. Discussion

In this study, we investigated the biological activity of tatridin A, isolated from the aerial parts of *Podanthus mitiqui*, and its derivative desacetyl-β-cyclopyrethrosin, a sesquiterpene lactone of the eudesmanolide type. Tatridin A exhibited significant cytotoxic effects against the advanced prostate cancer cell lines DU-145 and 22Rv1, whereas desacetyl-β-cyclopyrethrosin displayed markedly lower activity. Tatridin A is a germacranolide-type sesquiterpene lactone previously reported to induce apoptosis in human myeloid leukemia cell lines HL-60 and U937, mediated by early cytochrome c release, caspase-3 activation, and poly (ADP-ribose) polymerase-1 (PARP-1) cleavage [24]. In gastric cancer KATO III cells, tatridin A reduces invasiveness by antagonizing PGK1, an oncoprotein that promotes proliferative and anti-apoptotic signaling cascades, along with the downregulation of chemokine receptor 4 (CXCR4) and β-catenin [25].

Real-time cell death assays performed on DU-145 cells and the 22Rv1 cells revealed that tatridin A induces twice as much cell death after 48 h of incubation compared to desacetyl-β-cyclopyrethrosin (Figure 2a). This difference in potency was further reflected in the IC_50_ values, which were 81.38 μM for DU-145 and 50.67 μM for 22Rv1, compared with 166.9 μM and 290.3 μM, respectively, for desacetyl-β-cyclopyrethrosin (Figure 2b). According to Rivero et al. (2003), the cytotoxic effect of tatridin A was observed at concentrations of 9.8 ± 2.4 µM and 15.6 ± 2.3 µM in HL-60 and U937 leukemia cells, respectively [24]. Further studies by Ferraro et al. (2023) reported EC_50_ values of 38 ± 2 μM in KATO III gastric cancer cells and 18 ± 4 μM in THP-1 monocytic leukemia cells [25]. However, confluence analysis of DU-145 and 22Rv1 cells using IncuCyte^®^ revealed a dose-dependent effect of tatridin A at 48 h, which at low concentrations acted as a cytostatic agent by inhibiting cell proliferation without inducing cell death (Figure 3a). This behavior was further confirmed through clonogenic assays, which revealed IC_50_ values of 7.7 μM in DU-145 cells and 5.24 μM in 22Rv1 cells (Figure 3b,c), concentrations that effectively prevented colony formation. These findings are consistent with previous observations from our laboratory for other germacranolides found in *Podanthus mitiqui* such as erioflorin and erioflorin acetate [69] and highlight the activity of this class of sesquiterpene lactones as both cytotoxic and cytostatic agents. Notably, other germacranolides such as costunolide also inhibit proliferation in both hormone-dependent (LNCaP) and hormone-independent (DU-145 cells, PC-3) PC cell lines at low concentrations (IC_50_ range of 3–5.9 µM), demonstrating their ability to suppress cell division without inducing overt toxicity [68,70].

The difference in potency between tatridin A and its derivative desacetyl-β-cyclopyrethrosin were also noted in other compounds. For example, Rivero et al. studied the cytotoxic effect of several germacranolides including tatridin A, diacetyl tatridin A, tamirin, and ineupatorolide alongside the eudesmanolide reynosin in HL-60 and U937 leukemia cells. Among all compounds tested, reynosin was the least potent, requiring at least twice the concentration to achieve the same apoptotic effect as the germacranolides [24]. These results underscore the structural importance of germacranolides in preserving antiproliferative activity [71,72], with the α-methylene-γ-lactone moiety and specific stereochemical configurations being critical for cytotoxic activity against human cancer cells, as widely reported [73,74,75,76].

This study confirms that structural modification, such as the acid-mediated conversion by CDCl_3_ into a eudesmanolide derivative, significantly compromises the anticancer potential of tatridin A [16,19]. Germacranolides and eudesmanolides are structurally distinct sesquiterpene lactones. Germacranolides possess a flexible 10-membered carbocyclic ring with conjugated double bonds, whereas eudesmanolides are formed via additional cyclization, resulting in rigid bicyclic six-membered ring systems [77]. This structural distinction substantially impacts their molecular interactions with key proteins involved in biological pathways [16]. It is important to clarify that all biological assays were performed using purified and fully characterized samples of tatridin A (1) and desacetyl-β-cyclopyrethrosin (2), and no interconversion between the two compounds was detected in DMSO stock solutions or in cell culture media under the experimental conditions employed. The rearrangement of 1 into 2 was only observed in CDCl_3_ containing trace amounts of HCl, as described. Therefore, the loss of activity reported here is not related to instability during the cell-based assays but rather to a solvent-induced chemical transformation. Nevertheless, the acid sensitivity of tatridin A raises relevant questions for its pharmacological development, particularly regarding its potential oral administration, since acidic environments such as gastric fluid or tumor microenvironments could compromise its stability. These aspects were beyond the scope of the present work but warrant further investigation to fully assess the therapeutic applicability of this compound. A well-documented example of the impact of chemical transformations on drug efficacy is the deactivation of platinum-based anticancer drugs in DMSO solutions, which results from ligand exchange and the subsequent loss of pharmacological activity [78]. Our results reveal a comparable scenario for tatridin A, whose acid-catalyzed rearrangement into desacetyl-β-cyclopyrethrosin by CDCl_3_ is associated with a marked reduction in cytotoxic and antiproliferative activity against PC cells. This finding underscores the notion that chemical stability is a critical determinant of the biological potential of sesquiterpene lactones. The acid sensitivity of tatridin A may be particularly relevant in biological contexts characterized by acidic microenvironments. In such settings, the pharmacological profile of tatridin A could be compromised, thereby limiting its therapeutic applicability. Nonetheless, eudesmanolides have demonstrated potential anticancer activity in some cases. For instance, five eudesmanolides isolated from *Tanacetum vulgare* exhibited cytotoxic activity in human lung carcinoma A549 cells, with IC_50_ values ranging from 15.3 to 59.4 μM after 72 h of exposure [79]. Similarly, eudesmanolides from *Inula racemosa* showed cytotoxic effect effects against BEL-7402 (liver), HCT-8 (colon), A549 (lung), MCF-7 (breast), and HL-60 (leukemia) cancer cell lines, with moderate activity reported (IC_50_ between 10–50 µM after 96 h), although none exhibited anti-inflammatory activity [80]. Despite their reported cytotoxicity, eudesmanolides are generally considered to be less potent [81].

To elucidate the molecular mechanisms underlying the cytotoxic and cytostatic effects of tatridin A, we evaluated its impact on redox balance and ΔΨm in DU-145 and 22Rv1 cells. Desacetyl-β-cyclopyrethrosinitle was included to determine whether the derivative retained any of the original biological activity. It should be noted that the 50 μM concentration corresponds approximately to the IC_50_ of tatridin A but not of desacetyl-β-cyclopyrethrosin. This concentration was deliberately chosen to enable direct comparison of mechanistic endpoints under standardized conditions, while cytotoxic assays at sub-IC_50_ levels further supported the antiproliferative activity of both compounds. Our analysis showed that tatridin A and desacetyl-β-cyclopyrethrosin progressively increased total ROS production in both cell lines (Figure 4). However, tatridin A caused a rapid dissipation of ΔΨm within the first 10 h (Figure 5), unlike the derivative. These findings are consistent with the hypothesis that tatridin A may promote cell death through mitochondrial dysfunction.

In summary, tatridin A and desacetyl-β-cyclopyrethrosin enhanced cellular oxidative stress, but membrane potential dissipation occurred only in the presence of tatridin A. Similar effects have been reported for structurally related sesquiterpene lactones such as parthenolide, costunolide, and helenalin, which induce ROS accumulation and mitochondrial depolarization in PC and other tumor models [72,82,83,84,85]. To our knowledge, the present study is the first to describe such mechanistic effects for unmodified tatridin A, highlighting the novelty and significance of our findings. This observation is consistent with previous reports indicating that some germacranolides induce apoptosis by disrupting cellular redox balance [22,86,87,88]. The disparity may be due to the position of the functional group. It is proposed that the α-methylene-γ-lactone moiety in tatridin A favors covalent interactions with thiol residues in key mitochondrial proteins, a feature absent in the derivative. This may account for its enhanced ability to affect proteins that regulate mitochondrial potential homeostasis, ultimately leading to mitochondrial permeability transition pore (mPTP) opening and membrane potential collapse, as reported for costunolide [89]. In contrast, the more rigid structure of the eudesmanolide derivative may significantly hinder or prevent such interactions.

NF-κB activity was assessed in THP-1 reporter cells stimulated with LPS in the presence or absence of tatridin A and desacetyl-β-cyclopyrethrosin. Tatridin A inhibited NF-κB activation in a dose-dependently (Figure 6A). Under identical experimental conditions, tatridin A exhibited substantially greater potency across all concentrations evaluated (5, 10, and 20 µM) (Figure 6B). Western blot assays confirmed that tatridin A inhibits IκBα phosphorylation and prevents activation of the p65/p50 (NF-κB1/RelA) complex, an effect not observed with the derivative (Figure 6C). Phosphorylation of IκBα is essential to release the p65/p50 complex, which is otherwise retained in the cytoplasm [90]. This complex is critical for the transcription of genes encoding cytokines, chemokines, adhesion molecules, and anti-apoptotic proteins [91].

In this context, germacranolide-type sesquiterpene lactones such as parthenolide and costunolide inhibit NF-κB signaling by attenuating the IKK activity and the IκBα protein itself. These molecules have demonstrated activity in murine models of renal fibrosis [92] and cystic fibrosis cells [93]. Notably, tatridin A inhibited IκBα phosphorylation more effectively than BAY 11-7082, a synthetic NF-κB inhibitor that directly targets IKK to prevent IκBα phosphorylation [94]. NF-κB pathway attenuation by tatridin A could be related to its effects on cellular redox balance. It has been reported that elevated ROS levels may hinder NF-κB activation by oxidizing cysteine residues in IκB, thereby preventing its degradation [95]. Our findings are consistent with this mechanism, although further studies would be required to confirm a direct link. This attenuation was considerably less pronounced for desacetyl-β-cyclopyrethrosin.

## 5. Conclusions

In conclusion, the molecular mechanisms described herein indicate that the germacranolide structure of tatridin A is fundamental to its biological activity against advanced PC cells. These findings represent a promising therapeutic lead that warrants further investigation in preclinical models. Although the IC_50_ values for tatridin A reported in this study are slightly higher than those in leukemia models, its ability to inhibit tumor cell growth in PC is clearly supported. A deeper understanding of its mechanism of action could contribute to the development of novel adjuvant therapies against advanced PC.

Conversely, this observation highlights the importance of monitoring structural stability when considering germacranolides as anticancer candidates, since even minor transformations can alter their ability to modulate key signaling pathways such as NF-κB. Taken together, our study provides a cautionary perspective: while tatridin A displays promising cytotoxic and cytostatic effects, its acid-mediated conversion into a less active derivative must be taken into account in preclinical evaluations and formulation strategies aimed at preserving its integrity.

## Figures and Tables

**Table 1:** NMR spectroscopic data (^1^H 500 MHz, ^13^C 125 MHz, acetone-d6) for tatridin A and desacetyl-β-cyclopyrethrosin.

	Tatridin A (1)				Desacetyl-β-Cyclopyrethrosin (2)		
Position	d(^1^H)/ppm	m (*J* (Hz))	d(^13^C)/ppm	Position	d(^1^H)/ppm	m (*J* (Hz))	d(^13^C)/ppm
1	2.23 (α)	dd (11.8, 6.2)	36.1	1	2.30 (α)	ddd (13.3, 5.3, 2.1)	35.5
	1.90 (β)	m			2.06 (β)	m	
2	1.95 (α)	m	28.6	2	1.60 (α)	dddd (13.6, 13.0, 11.5, 5.2)	32.4
	1.70 (β)	dddd (13.8, 11.1, 6.3, 2.4)			1.82 (β)	dddd (12.6, 5.2, 5.2, 2.1)	
3	4.42	ddd (11.1, 4.5, 4.3)	66.7	3	3.56	ddd (11.4, 5.0, 5.0)	78.4
4	-	-	143.6	4	-	-	43.6
5	5.27	d (10.4)	127.1	5	2.46 (α)	dd (11.6, 3.7)	41.3
					1.53 (β)	dd (11.9, 11.9)	
6	4.63	t (9.5)	75.1	6	4.04	ddd (12.3, 11.4, 3.8)	77.6
7	2.78	tt (8.9, 3.3)	53.3	7	2.60	dddd (11.3, 10.0, 3.1, 3.1)	55.5
8	4.51	ddd (10.7, 9.1, 4.5)	71.2	8	4.13	ddd (10.0, 10.0, 6.0)	67.9
9	4.96	d (10.6)	132.0	9	2.01	d (10.0)	57.8
10	-	-	133.8	10	-	-	145.2
11	-	-	140.4	11	-	-	140.1
12	-	-	170.4	12	-	-	170.7
13	6.16 (α)	dd (3.2, 1.7)	121.7	13	5.93 (α)	dd (3.0, 1.3)	118.7
	6.07 (β)	dd (3.5, 1.7)			5.98 (β)	dd (3.2, 1.3)	
14	1.77	3H, s (br)	15.6	14	4.98 (α)	q (1.4)	109.3
					4.83 (β)	q (1.4)	
15	1.79	3H, d (1.5)	17.1	15	0.84	3H, s	14.2
C^3^-O*H*	3.92	d (3.9)	-	C^3^-O*H*	3.87	d (5.2)	-
C^8^-O*H*	4.23	d (4.5)	-	C^8^-O*H*	3.74	d (6.0)	-

**Table 2:** Summary of IC_50_ values for cytotoxicity (Sytox Green assay), long-term survival (clonogenic assay), and net viability (combined proliferation and cytotoxicity).

Cell Line	Compound	IC_50_ (Sytox, µM)	IC_50_ (Net Viability, µM)	IC_50_ (Clonogenic, µM)
**DU-145**	Tatridin A	81.38 ± 2.7	53.66 ± 1.8	7.70 ± 1.4
**DU-145**	DesβCP	166.9 ± 3.2	101.20 ± 2.9	ND
**22Rv1**	Tatridin A	50.67 ± 1.9	35.38 ± 2.1	5.24 ± 0.95
**22Rv1**	DesβCP	290.3 ± 8.3	80.98 ± 3.7	ND

DesβCP = Desacetyl-β-cyclopyrethrosin; ND = not determined.

## Data Availability

The data underlying this study are available in the article and its Supporting Information. FAIR Data are available at https://zenodo.org/records/14652072 (accessed on 15 January 2025).

## Supplementary Materials

The following supporting information can be downloaded at: https://www.mdpi.com/article/10.3390/jox15050161/s1. Figure S1: Molecular structure of compounds 1 and 2 and comparison with 3. Compounds 2 and 3; Figure S2: NOESY correlations for tatridin A; Figure S3: HMBC correlations for tatridin A; Figure S4: 1H NMR (500 MHz, acetone-d6) of tatridin A; Figure S5: ^13^C ^1^H NMR (125 MHz, acetone-*d*_6_) of tatridin A; Figure S6: H, H-COSY (500 MHz, acetone-*d*_6_) of tatridin A; Figure S7: HSQC (500/125 MHz, acetone-*d*_6_) of tatridin A; Figure S8: HMBC (500/125 MHz, acetone-*d*_6_) of tatridin A; Figure S9: NOESY (500 MHz, acetone-*d*_6_) of tatridin A; Figure S10: NOESY correlations for desacetyl-β-cyclopyrethrosin; Figure S11: HMBC correlations for desacetyl-β-cyclopyrethrosin; Figure S12: ^1^H NMR (500 MHz, acetone-*d*_6_) of desacetyl-β-cyclopyrethrosin; Figure S13: ^13^C ^1^H NMR (125 MHz, acetone-*d*_6_) of desacetyl-β-cyclopyrethrosin; Figure S14: H,H-COSY (500 MHz, acetone-*d*_6_) of desacetyl-β-cyclopyrethrosin; Figure S15: HSQC (500/125 MHz, acetone-*d*_6_) of desacetyl-β-cyclopyrethrosin; Figure S16: HMBC (500/125 MHz, acetone-*d*_6_) of desacetyl-β-cyclopyrethrosin; Figure S17. NOESY (500 MHz, acetone-*d*_6_) of desacetyl-β-cyclopyrethrosin; Figure S18: Original Western blot images of of IκBα, phospho-IκBα and α-Tubulin reported in Figure 6c. Table S1: Comparative crystal data for compounds 1, 2 and 3; Table S2. NMR-data of tatridin A and comparison with literature data; Table S3: NMR-data of desacetyl-β-cyclopyrethrosin and comparison with literature data. File S1: Original images of Figure 5b. References [46,47,48,56] are cited in the Supplementary Material and/or in the main text.

## Abbreviations

The following abbreviations are used in this manuscript:

AR	Androgen receptor
CD_2_Cl_2_	Deuterated dichloromethane
CH_2_Cl_2_	Dichloromethane
DMSO	Dimethyl sulfoxide
EtOAc	Ethyl acetate
FBS	Fetal bovine serum
H_2_DCFDA	Dichlorodihydrofluorescein diacetate
IC_50_	Half maximal inhibitory concentration
LPS	Lipopolysaccharide
NF-κB	Nuclear transcription factor kappa B
NMR	Nuclear magnetic resonance
ORTEP	Oak Ridge Thermal Ellipsoid Plot
PC	Prostate cancer
ROS	Reactive oxygen species
SEAP	Secreted embryonic alkaline phosphatase
STAT3	Signal transducer and activator of transcription 3
TMRM	Tetramethylrhodamine methyl ester
