# Supplementary material for: The Antiproliferative Activity of Tatridin A Against Prostate Cancer Cells Is Lost in Acid Medium by Transformation to Desacetyl-β-Cyclopyrethrosin
Source: J Xenobiot. 2025 Oct 9;15(5):161. doi: 10.3390/jox15050161 (PMC12565412; doi:10.3390/jox15050161)
Supplement: Supplementary file 1 [file jox-15-00161-s001.zip › jox-3782928-supplementary.pdf]

Supporting Information

The Antiproliferative Activity of Tatridin A Against Prostate Cancer Cells Is Lost in Acid Medium by Transformation to Desacetyl-β-Cyclopyrethrosin

Cecilia Villegas, Rebeca Pérez, Camilo Céspedes-Méndez, Viviana Burgos, Ricardo Baggio, Sebastián Suárez, Bernd Schmidt and Cristian Paz

Single crystal structure analyses of tatridin A (1) and desacetyl-β-cyclopyrethrosin (2)

The crystal and molecular structure of compounds **1** and **2** (**Scheme 1**) have been determined by single crystal X-ray diffraction. **Figure 1** in the main manuscript shows ellipsoid plots for both molecules, drawn at 40% probability level, and **Table S1** presents relevant crystal and refinement data. Bond distances and angles are unexceptional. Complete crystallographic data sets have been deposited in CIF format at the CSD, as deposition numbers 2423080 (Structure **2**) and 2423081 (Structure **1**).

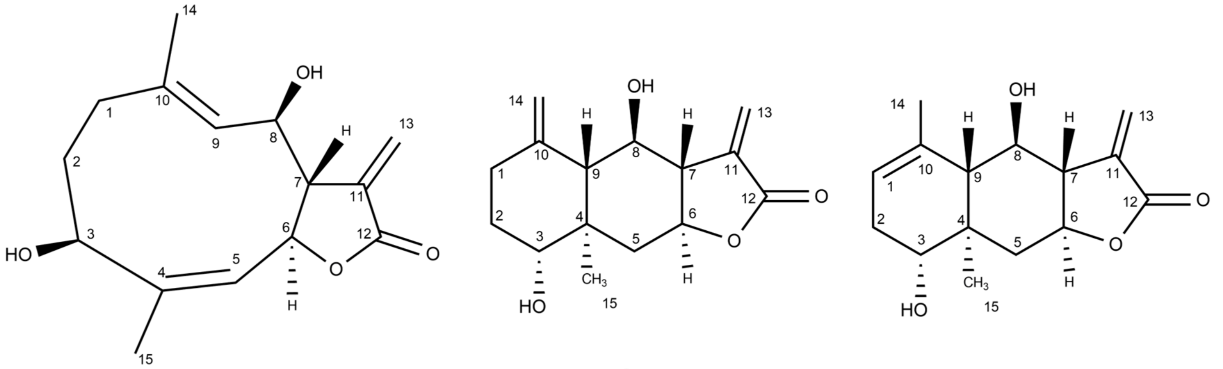

1 **2** **3**

Figure S1. Molecular structure of compounds **1** and **2** and comparison with **3**. Compounds **2** and **3** differ in the position of the double bond at C10.

Compound **1** (Tatridin, $C_{15}H_{20}O_4$•H_2O) crystallizes in the chiral space group $P3_22$, with two independent molecules per asymmetric unit. The structure has already been reported in the literature (CSD code EMUHUC), where the absolute conformation was determined through the use of the Cotton effect (chiral centers: 3*S*, 4*R*, 9*S*, 12*S*) [1]. However, the X-ray analysis therein reported wrongly assigned the structure to space group $P3_1$, with four independent molecules in the asymmetric unit. In addition, the chirality in the crystallographic model presented was inverted with regard to the one determined in the same work by the Cotton effect. We have thus solved and refined the structure in the correct space group $P3_22$, which led to the correct handedness. The hydration water molecule, however, appeared smeared out in the difference maps, for what we refined the structure as having no solvates, and resorted to the SQUEEZE procedure implemented in PLATON, to account for the lacking electron density.

Compound **2** ($C_{15}H_{20}O_4$) crystallizes in the chiral space group $P2_12_12_1$, with one molecule per asymmetric unit. Bond distances and angles confirm the structure shown in **Figure S1**, with C12=O2, C11=C13 and C10=C14 being exocyclic double bonds. In this regard, it is relevant to distinguish compound **2** from a reported, almost isostructural relative **3** (CSD entry LAHDIJ, no bibliographic reference available, see **Figure S1**) where the double bond C10=C14 appears shifted towards C1=C10, inside the ring, the methylene unit in compound **2** becoming a methyl group in compound **3**. **Table S1** gives a comparison of crystal data for both species. It is worth mentioning that refinement of the Cu Kα X-ray diffraction data in compound **2** unambiguously determines the configuration of its chiral centers as 3*R*,4*R*,6*S*,7*S*,8*R*,9*S*. Structure **3** (CSD entry LAHDIJ, no bibliographic reference available) [2], instead, has been reported in an inverted handedness.

References:

1. Turdybekov KM, Morozova OA, Ivasenko SA, Makhmutova AS, Turdybekov DM, Adekenov SM. Molecular Structure and Absolute Configuration of Tatridin A and B from Lepidolopha karatavica. Chemistry of Natural Compounds. 2021;57(1):83-7.

2. Abduazimov BK, Tashkhodzhaev B, Nasirov S, Sham'yanov ID, Yagudaev MR, Malikov VM, et al. Structure of mucrolide. Chemistry of Natural Compounds. 1991;27(1):15-9. https://doi.org/10.1007/BF00629823.

Table S1. Comparative crystal data for compounds **1**, **2** and **3**

**Compound 1** (this work)	**Compound 2** (this work)	**Compound 3** (LAHDIJ)
$C_{15}H_{20}O_4$•H_2O	$C_{15}H_{20}O_4$	$C_{15}H_{20}O_4$
M_r = 282.32	M_r = 264.31	M_r = 264.31
Trigonal, P3_22	Orthorhombic, $P2_12_12_1$	Orthorhombic, $P2_12_12_1$
a = 15.83340(10) Å	a = 8.40944 (10) Å	a = 8.296 (2) Å
b = 15.83340(10) Å	b = 12.17498 (13) Å	b = 12.400 (4) Å
c = 22.0848(2) Å	c = 13.62452 (17) Å	c = 13.366 (4) Å
V = 4794.82(7) Å^3	V = 1394.94 (3) Å^3	V = 1374.97 Å^3
Z = 12	Z = 4	Z = 4
F(000) = 1824	F(000) = 568	F(000) = 568
D_x = 1.173 gcm^{-3}	D_x = 1.259 gcm^{-3}	D_x = 1.277 gcm^{-3}
Cu Kα radiation, λ = 1.54184 Å	Cu Kα radiation, λ = 1.54184 Å	Mo Kα radiation, λ = 0.71073 Å
μ = 0.72 mm^{-1}	μ = 0.74 mm^{-1}	μ = 0.09 mm^{-1}
T = 295 K	T = 295 K	
Fragment, colourless	Fragment, colourless	
0.32 × 0.20 × 0.14 mm	0.40 × 0.28 × 0.18 mm	

NMR-spectroscopical data and copies of spectra for tatridin A

Table S2. NMR-data of tatridin A and comparison with literature data.

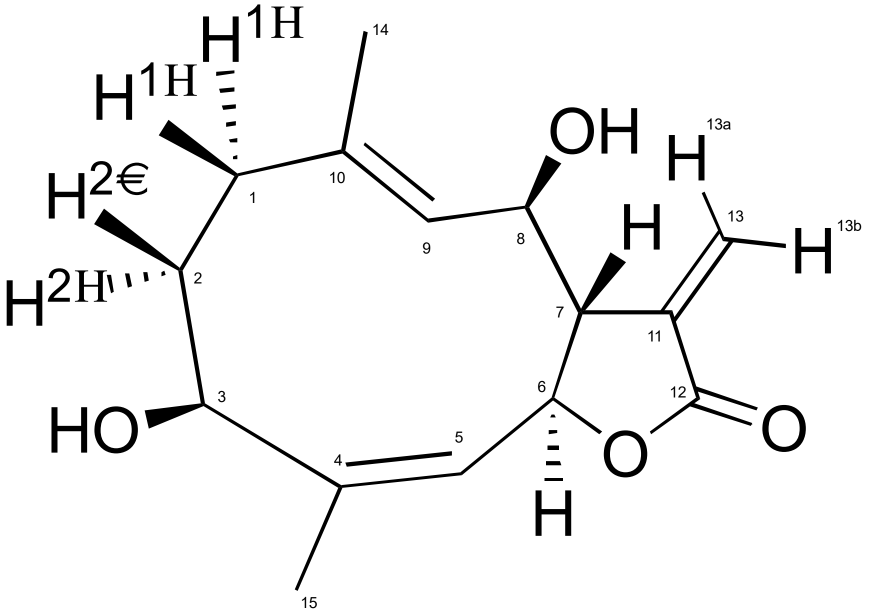

	This work[a]			Literature data for comparison[b]		
position	δ(^{1}H)/ppm	m (J (Hz))	δ(^{13}C)/ppm	δ(^{1}H)/ppm	m/J (Hz)	δ(^{13}C)/ppm
1	2.23 (α)<br>1.90 (β)	dd (11.8, 6.2)<br>m	36.1	2.28<br>1.92	q (6.0)<br>m	35.83
2	1.95 (α)<br>1.70 (β)	m<br>dddd (13.8, 11.1, 6.3, 2.4)	28.6	1.98<br>1.74	m<br>m	27.87
3	4.42	ddd (11.1, 4.5, 4.3)	66.7	4.36	dd (10.5, 5.0)	66.8
4	--	--	143.6	--	--	143.43
5	5.27	d (10.4)	127.1	5.31	d (9.0)	126.93
6	4.63	t (9.5)	75.1	4.65	t (9.0)	75.89
7	2.78	tt (8.9, 3.3)	53.3	2.80	m	52.94
8	4.51	ddd (10.7, 9.1, 4.5)	71.2	4.45	dd (10.0, 9.0)	70.95
9	4.96	d (10.6)	132.0	4.96	d (10.0)	131.1
10	--	--	133.8	--	--	134.37
11	--	--	140.4	--	--	139.00
12	--	--	170.4	--	--	171.91
13	6.16 (H^{13a})<br>6.07 (H^{13b})	dd (3.2, 1.7)<br>dd (3.5, 1.7)	121.7	6.24<br>6.23	d (2.0)<br>d (2.0)	123.53
14	1.77	3H, s (br.)	15.6	1.79	3H, s	15.54
15	1.79	3H, d (1.5)	17.1	1.82	3H, s	17.02
C^3-O*H*	3.92	d (3.9)	--	not reported		
C^8-O*H*	4.23	d (4.5)	--	not reported		

[a] ^{1}H NMR (500 MHz, acetone-d_6); ^{13}C NMR (125 MHz, acetone-d_6). [b] ^{1}H NMR (600 MHz, $CDCl_3$); ^{13}C NMR (150 MHz, $CDCl_3$): Turdybekov, K. M.; Morozova, O. A.; Ivasenko, S. A.; Makhmutova, A. S.; Turdybekov, D. M.; Adekenov, S. M. Molecular Structure and Absolute Configuration of Tatridin A and B from *Lepidolopha karatavica*. Chem. Nat. Compd**.** 2021, 57, 83-87.

.

Figure S2. NOESY correlations for tatridin A

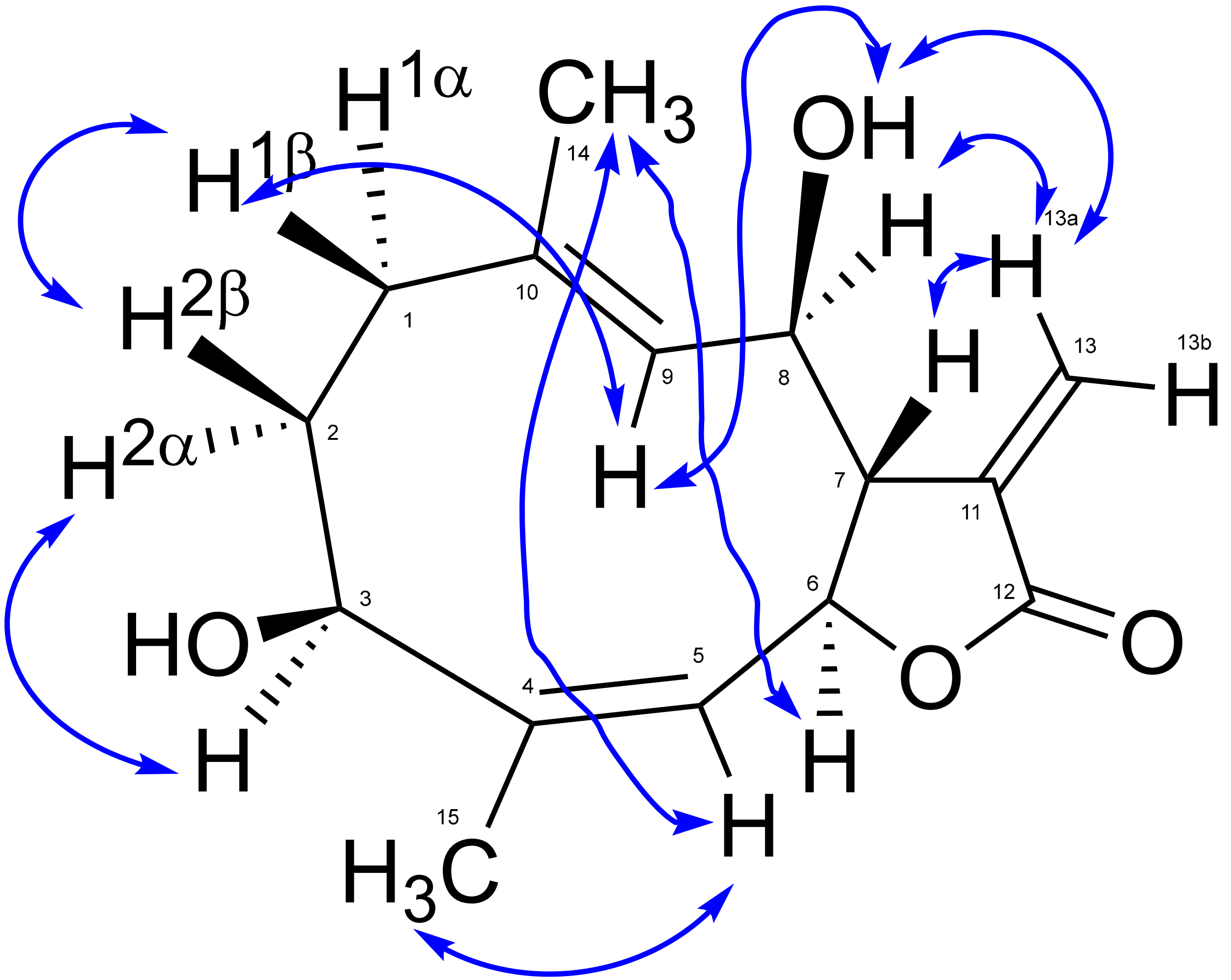

Figure S3. HMBC correlations for tatridin A.

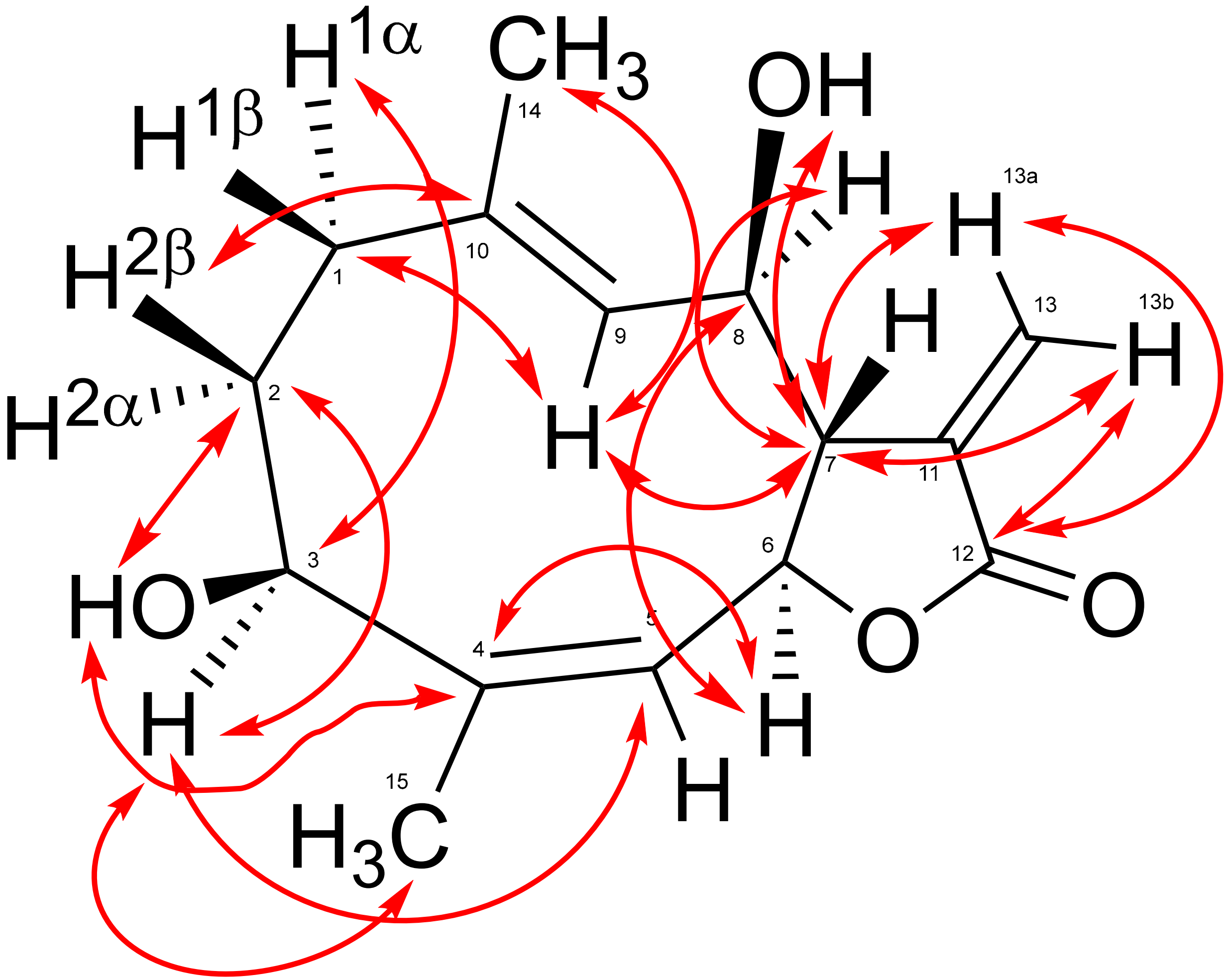

Figure S4. ^{1}H NMR (500 MHz, acetone-d_6) of tatridin A.

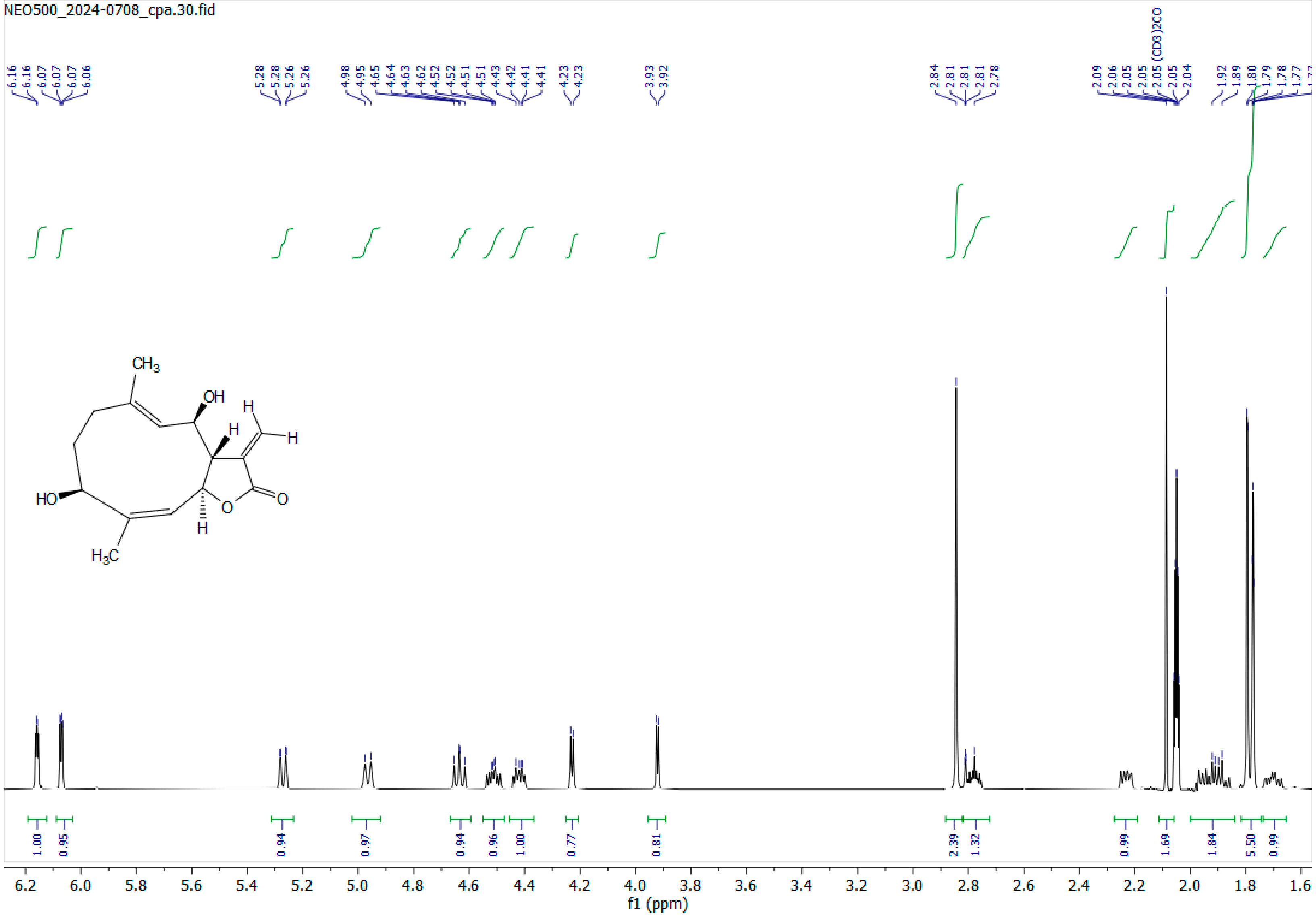

Figure S5. ^{13}C ^{1}H NMR (125 MHz, acetone-d_6) of tatridin A.

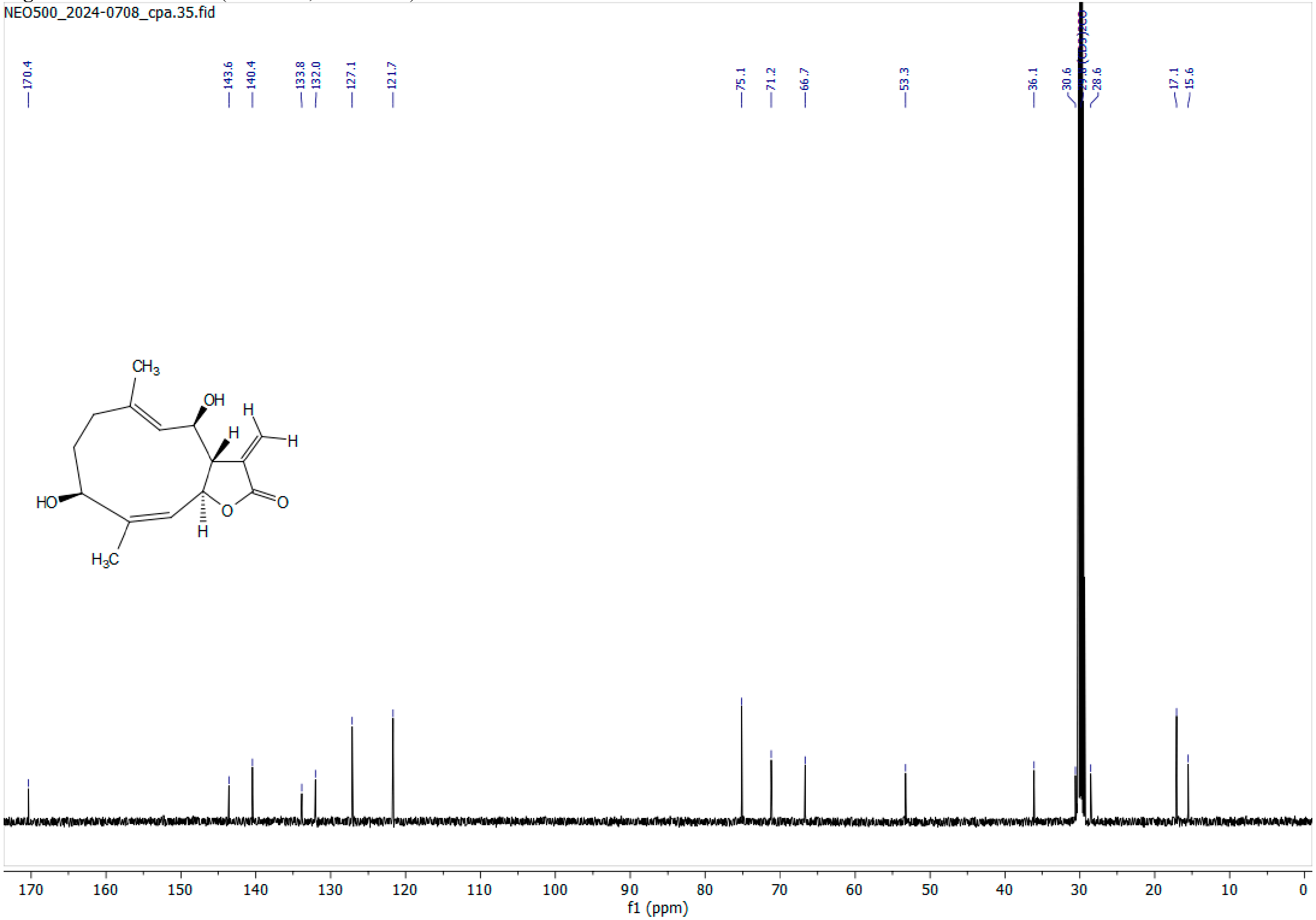

Figure S6. H, H-COSY (500 MHz, acetone-d_6) of tatridin A.

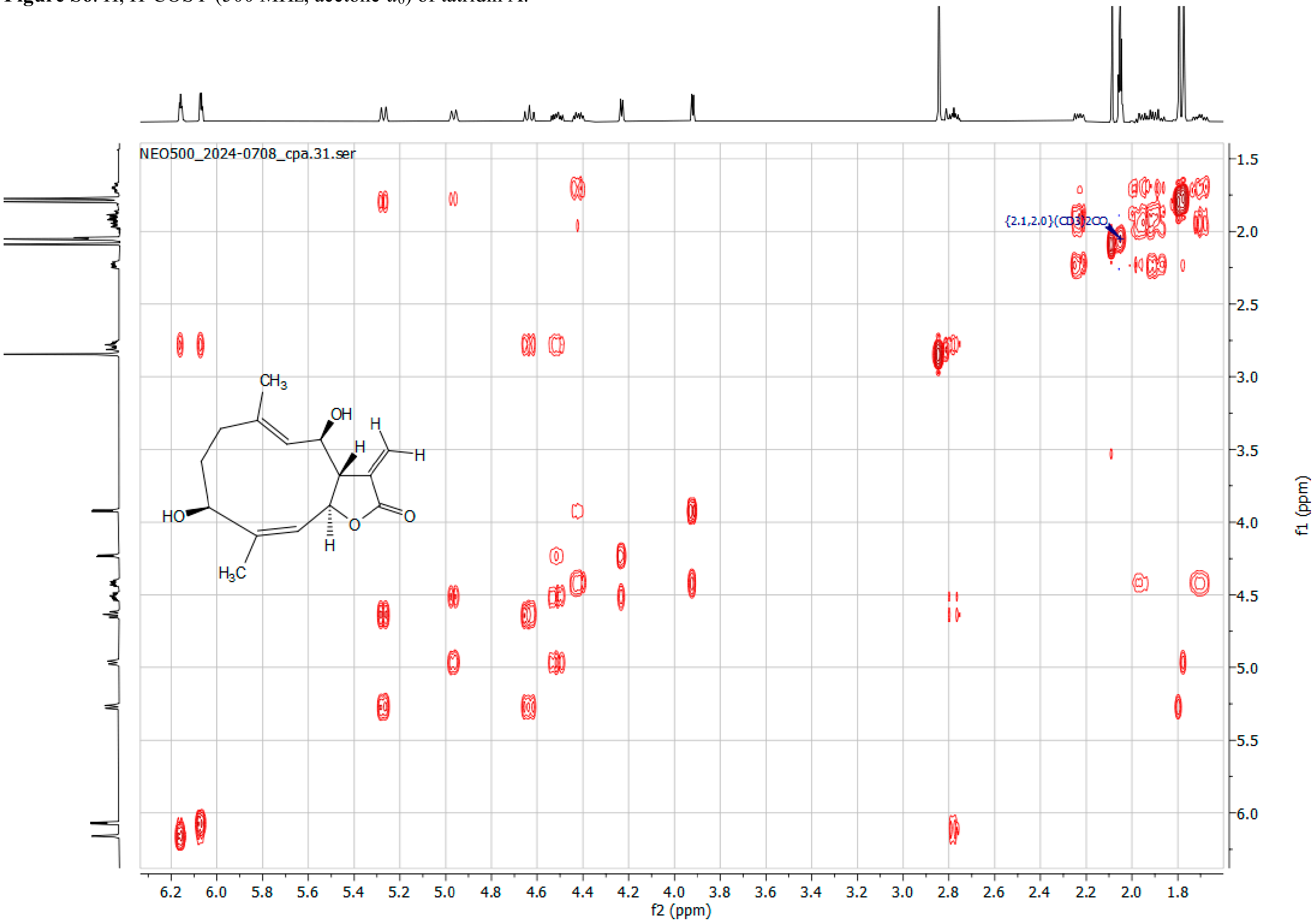

Figure S7. HSQC (500/125 MHz, acetone-d_6) of tatridin A.

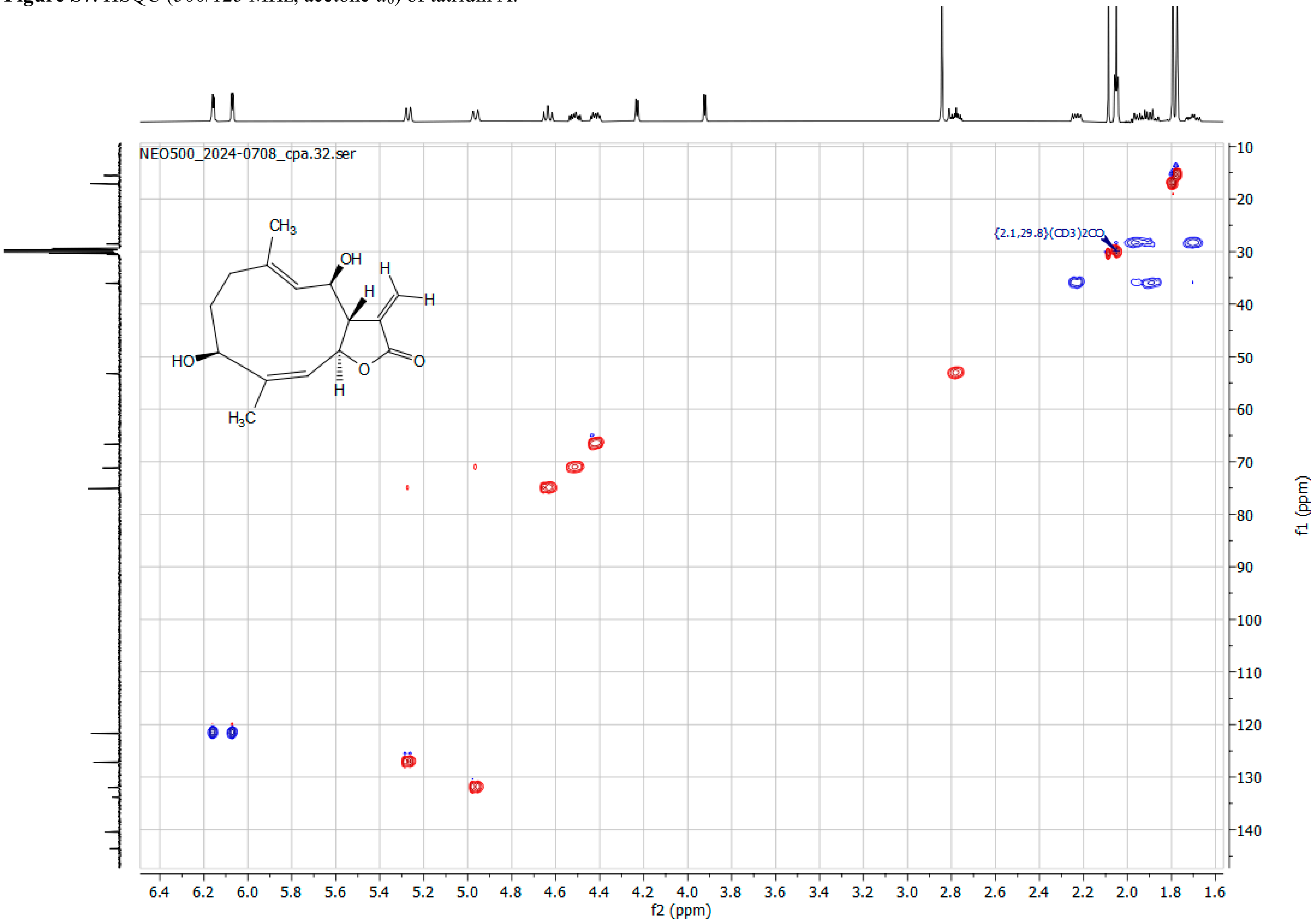

Figure S8. HMBC (500/125 MHz, acetone-d_6) of tatridin A.

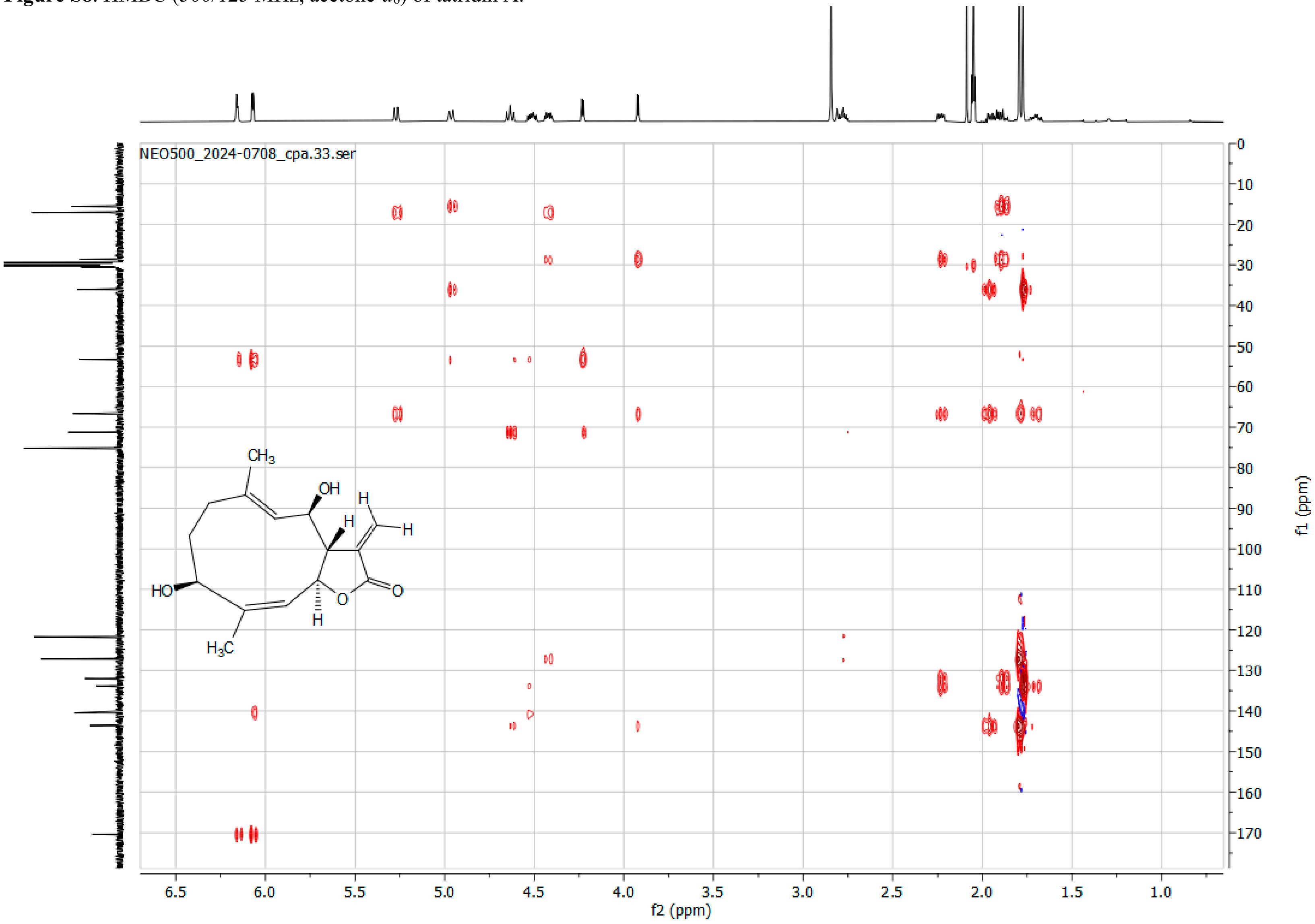

Figure S9. NOESY (500 MHz, acetone-d_6) of tatridin A.

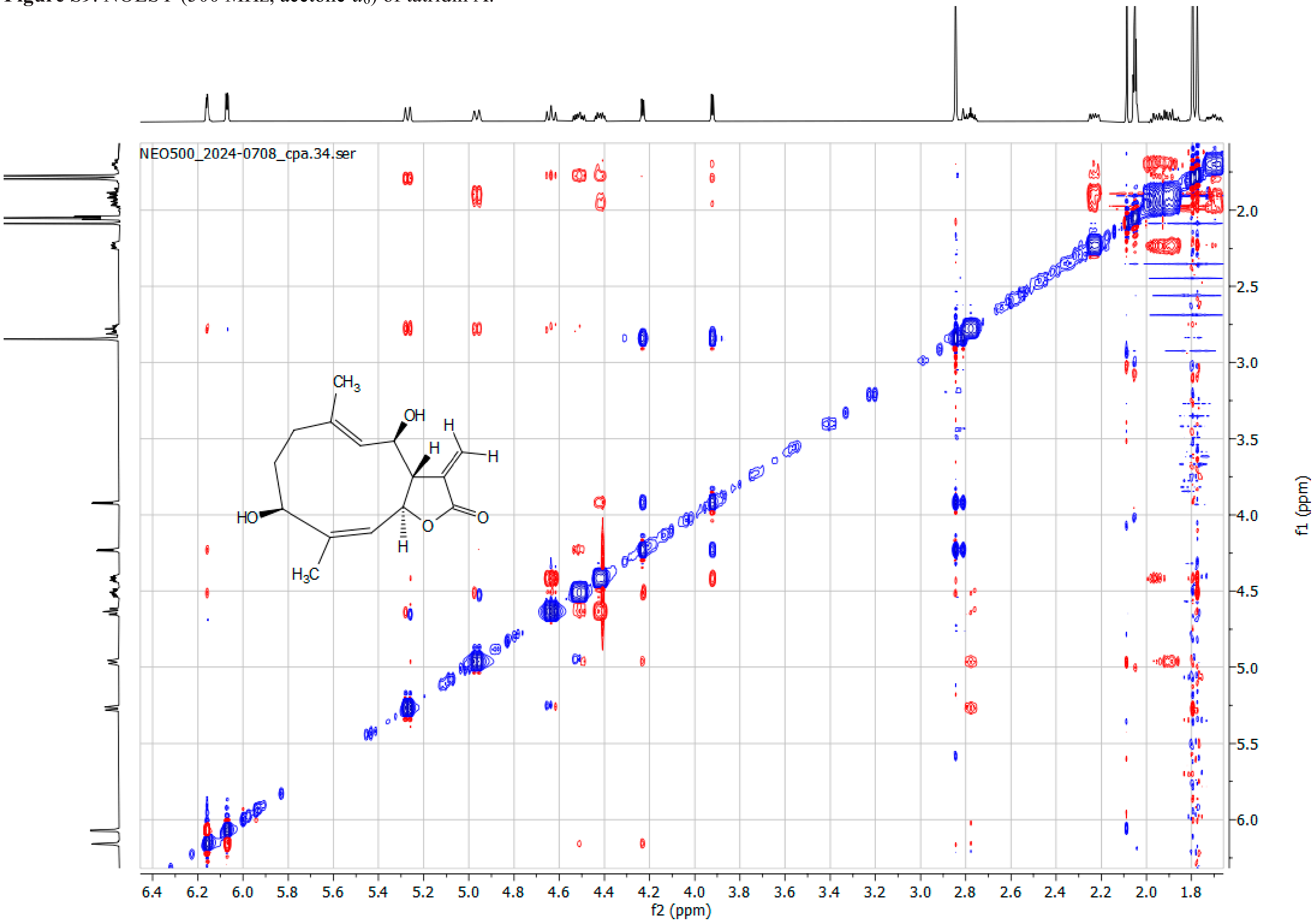

NMR-spectroscopical data and copies of spectra for desacetyl-β-cyclopyrethrosin

Table S3. NMR-data of desacetyl-β-cyclopyrethrosin and comparison with literature data.

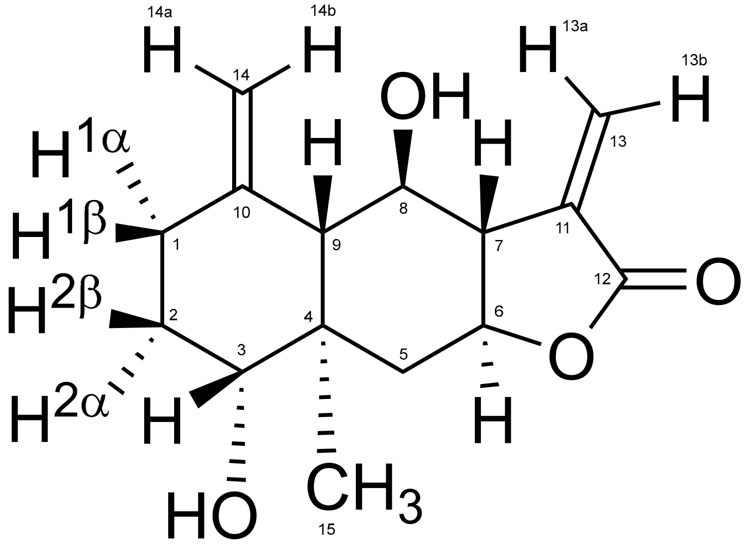

	This work[a]			Literature data for comparison[b]		
position	δ(^{1}H)/ppm	m (*J* (Hz))	δ(^{13}C)/ ppm	δ(^{1}H)/ppm	m/*J* (Hz)	δ(^{13}C)/ ppm
1	2.30 (α)<br>2.06 (β)	ddd (13.3, 5.3, 2.1)<br>m	35.5	2.23<br>1.8-2.1	ddd (12.1, 5.2, 2.0)<br>m	34.5
2	1.60 (α)<br>1.82 (β)	dddd (13.6, 13.0, 11.5, 5.2)<br>dddd (12.6, 5.2, 5.2, 2.1)	32.4	1.4-1.6<br>1.78	m<br>dddd (15.4, 5.2, 4.8, 2.4)	31.5
3	3.56	ddd (11.4, 5.0, 5.0)	78.4	3.48	dd (11.0, 4.8)	78.3
4	--	--	43.6			42.8
5	2.46 (α)<br>1.53 (β)	dd (11.6, 3.7)<br>dd (11.9, 11.9)	41.3	2.42<br>1.43	dd (11.7, 3.6)<br>t (11.8)	40.3
6	4.04	ddd (12.3, 11.4, 3.8)	77.6	3.92	td (11.5, 3.6)	76.4
7	2.60	dddd (11.3, 10.0, 3.1, 3.1)	55.5	2.40	ddt (11.5, 10.0, 3.0)	54.0
8	4.13	ddd (10.0, 10.0, 6.0)	67.9	4.04	br. t (9.8)	67.3
9	2.01	d (10.0)	57.8	1.90	br. d (11.5)	57.2
10	--	--	145.2	--	--	144.2
11	--	--	140.1	--		137.3
12	--	--	170.7	--		170.7
13	5.93 (H^{13a})<br>5.98 (H^{13b})	dd (3.0, 1.3)<br>dd (3.2, 1.3)	118.7	5.94<br>5.88	dd (3.0, 1.0)<br>dd (3.0, 1.0)	118.7
14	4.98 (H^{14a})<br>4.83 (H^{14b})	q (1.4)<br>q (1.4)	109.3	4.91<br>4.73	d (1.1)<br>d (1.1)	108.7
15	0.84	3H, s	14.2	0.75	3H, s	13.8
C^3-O*H*	3.87	d (5.2)	--	not reported		--
C^8-O*H*	3.74	d (6.0)	--	not reported		--

[a] ^{1}H NMR (500 MHz, acetone-*d*$_6$); ^{13}C NMR (125 MHz, acetone-*d*$_6$). [b] ^{1}H NMR (200 MHz, $CDCl_3$/acetone-*d*$_6$): Cardona, M. L.; Fernández, I.; García, B.; Pedro, J. R. Revision of the Structure of an Eudesmanolide Isolated from *Lasiolaena santosii*. J. Nat. Prod. 1990, *53*, 1042-1045; ^{13}C NMR (100 MHz, $CDCl_3$): Konstantinopoulou, M.; Karioti, A.; Skaltsas, S.; Skaltsa, H. Sesquiterpene Lactones from *Anthemis altissima* and Their Anti-Helicobacter pylori Activity. J. Nat. Prod. 2003, 66, 699-702.

Figure S10. NOESY correlations for desacetyl-β-cyclopyrethrosin.

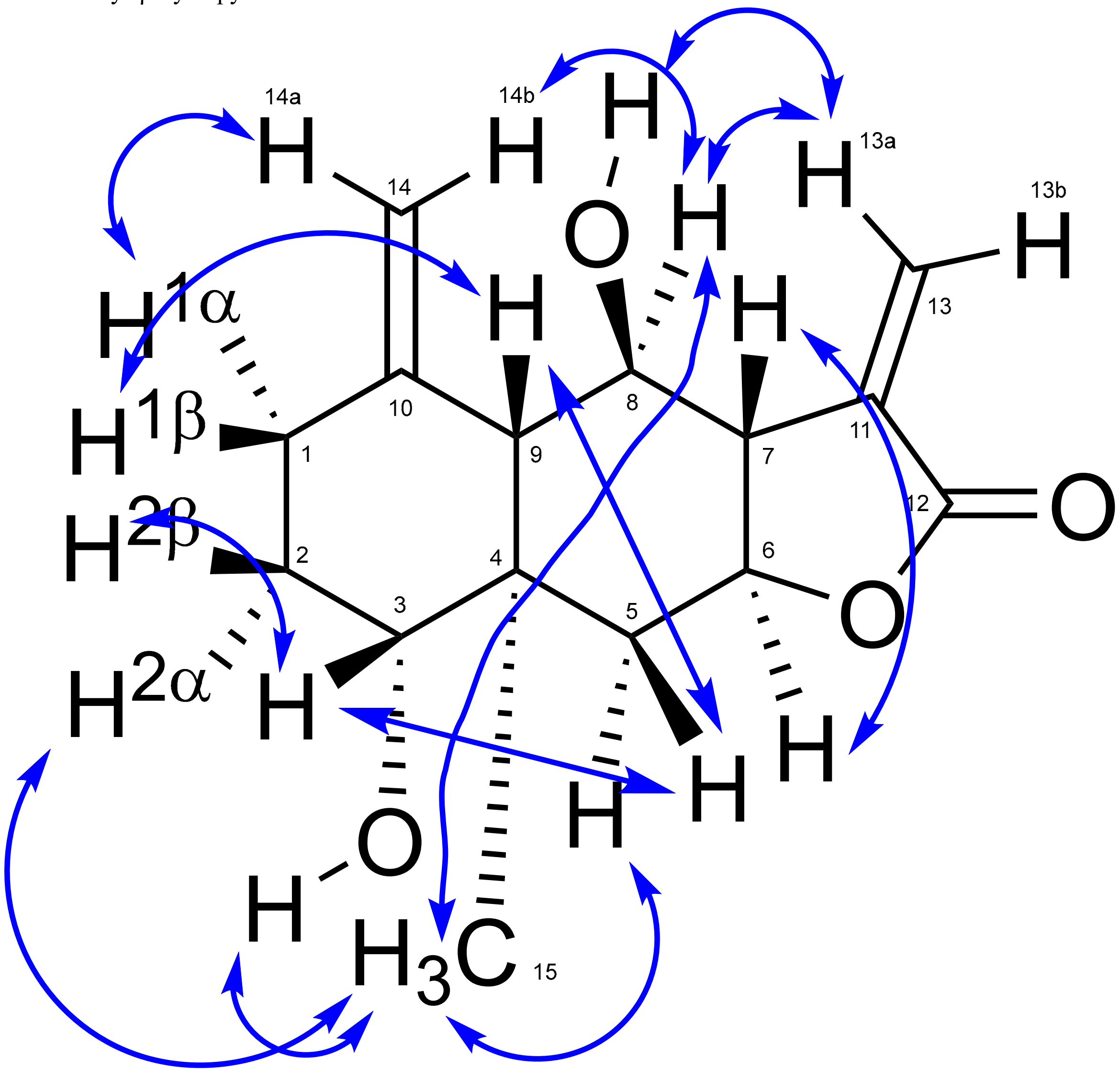

Figure S11. HMBC correlations for desacetyl-β-cyclopyrethrosin.

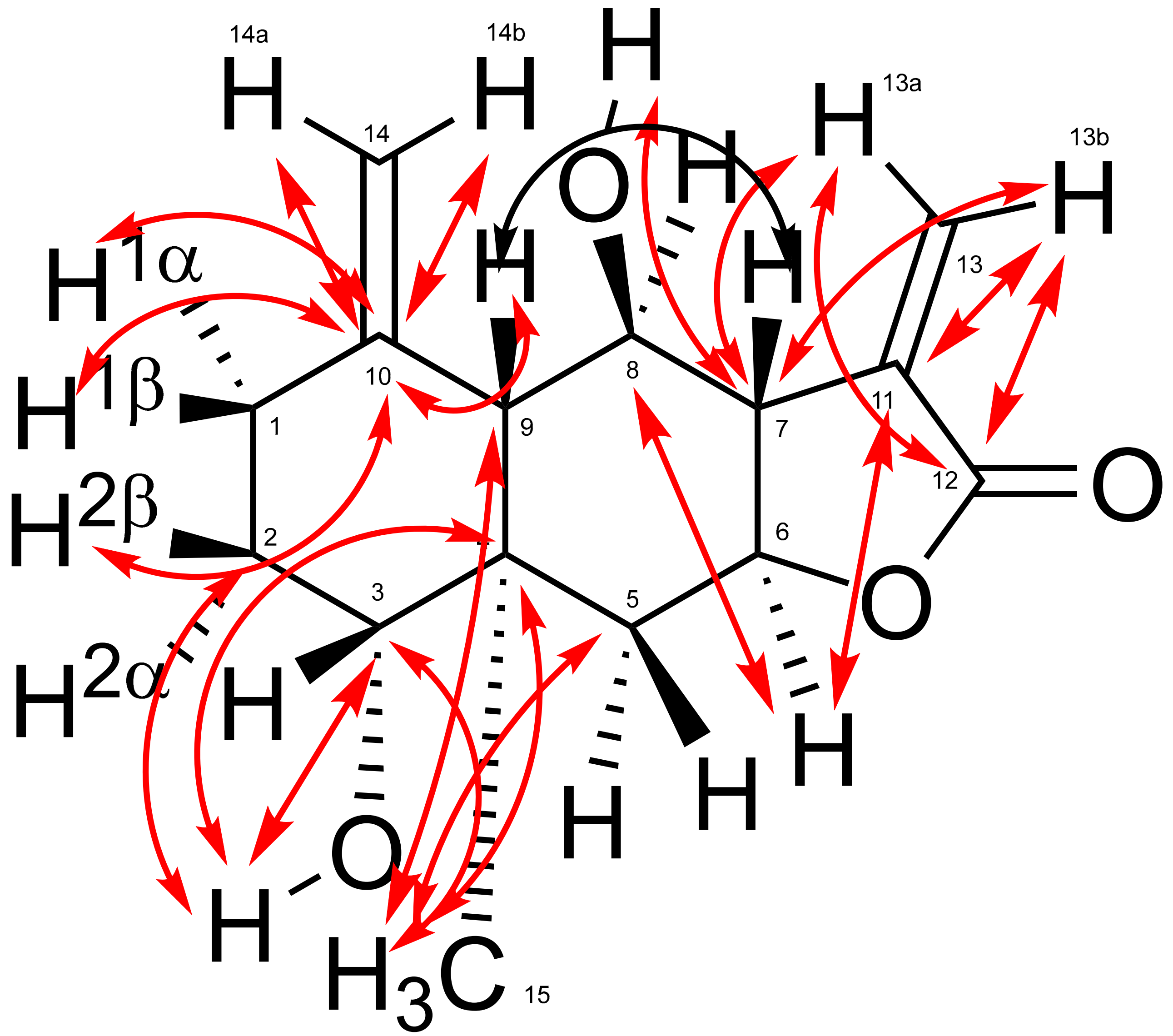

Figure S12. ^{1}H NMR (500 MHz, acetone-d_6) of desacetyl-β-cyclopyrethrosin.

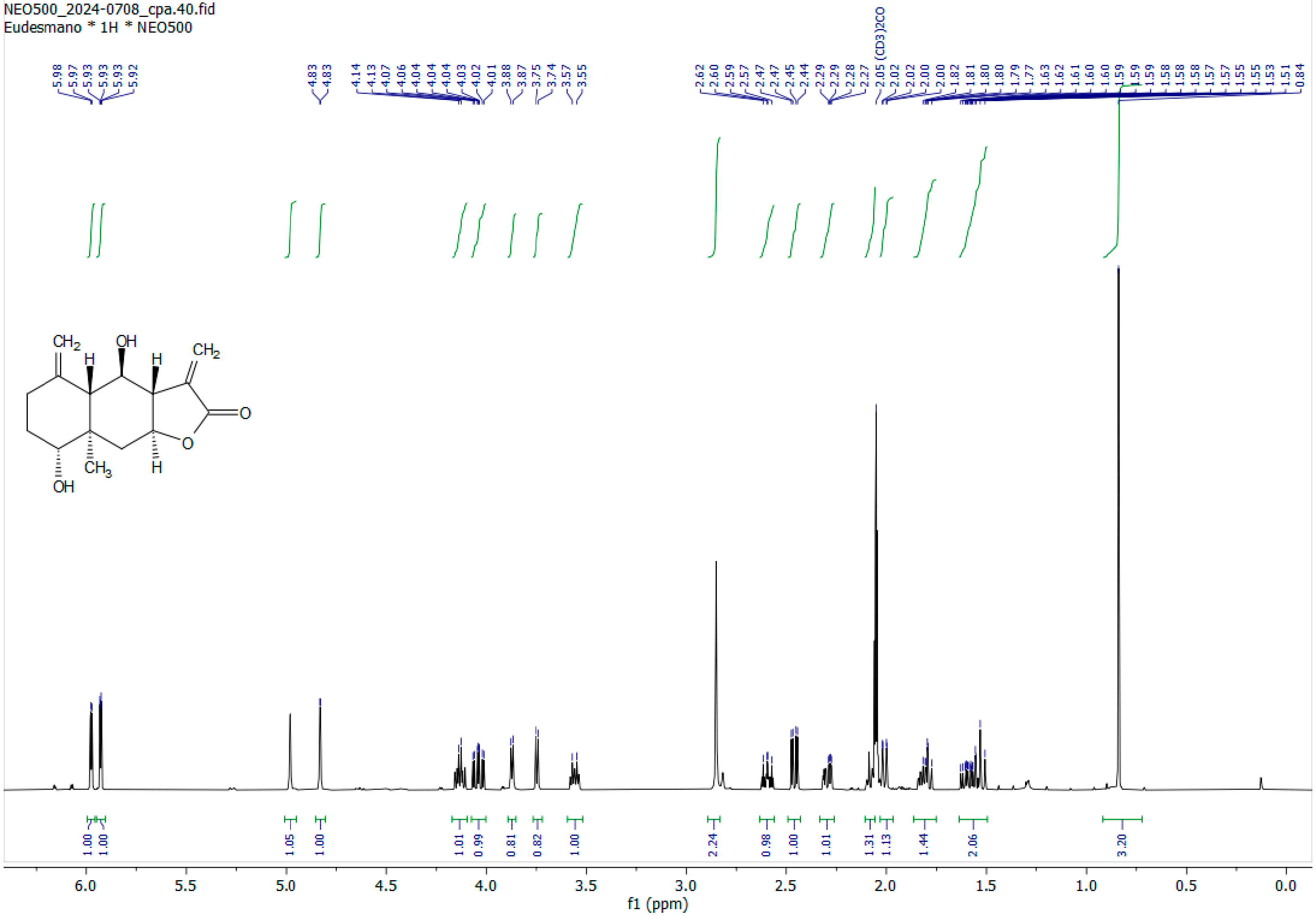

Figure S13. ^{13}C ^{1}H NMR (125 MHz, acetone-d_6) of desacetyl-β-cyclopyrethrosin.

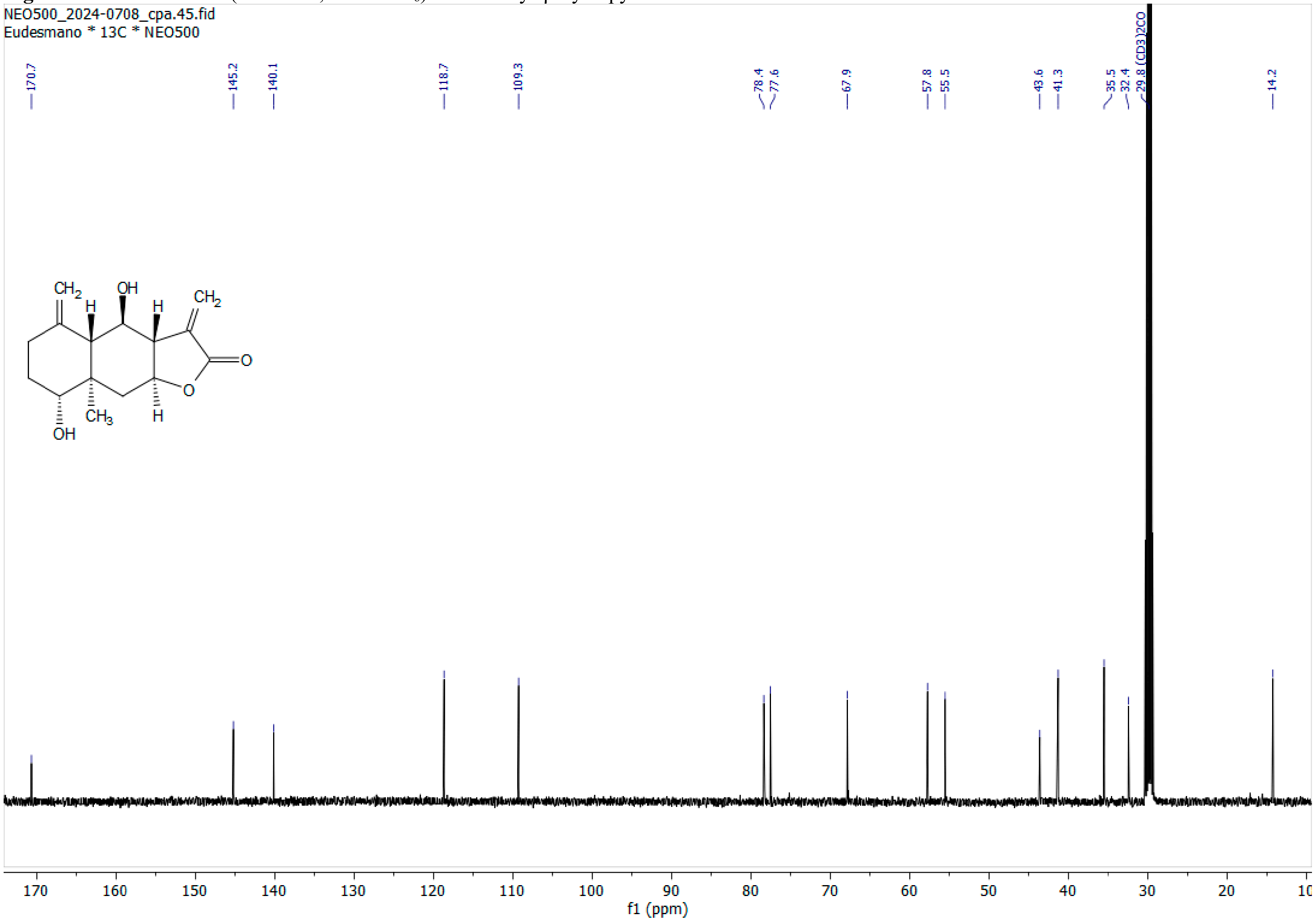

Figure S14. H,H-COSY (500 MHz, acetone-d_6) of desacetyl-β-cyclopyrethrosin.

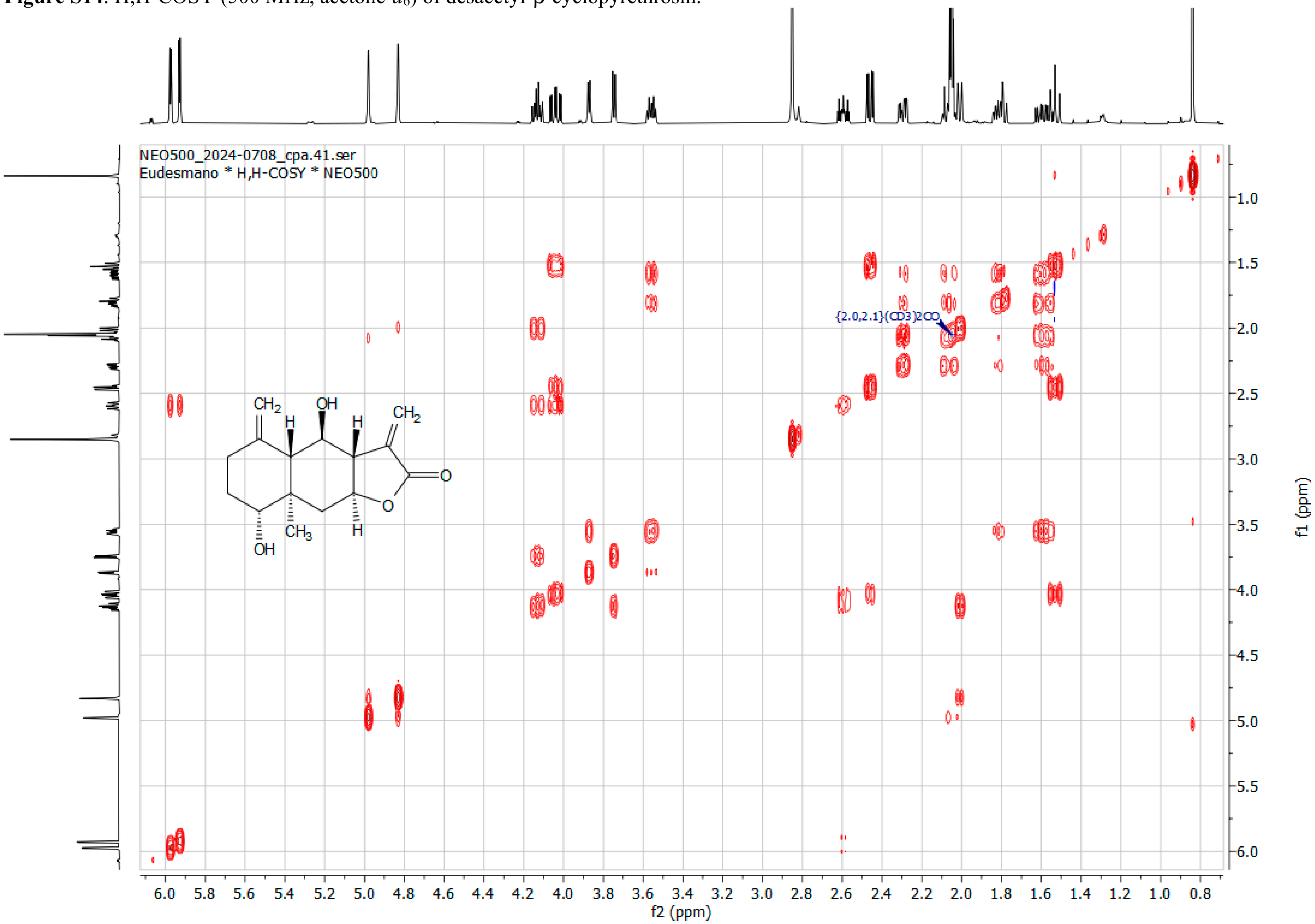

Figure S15. HSQC (500/125 MHz, acetone-d_6) of desacetyl-β-cyclopyrethrosin.

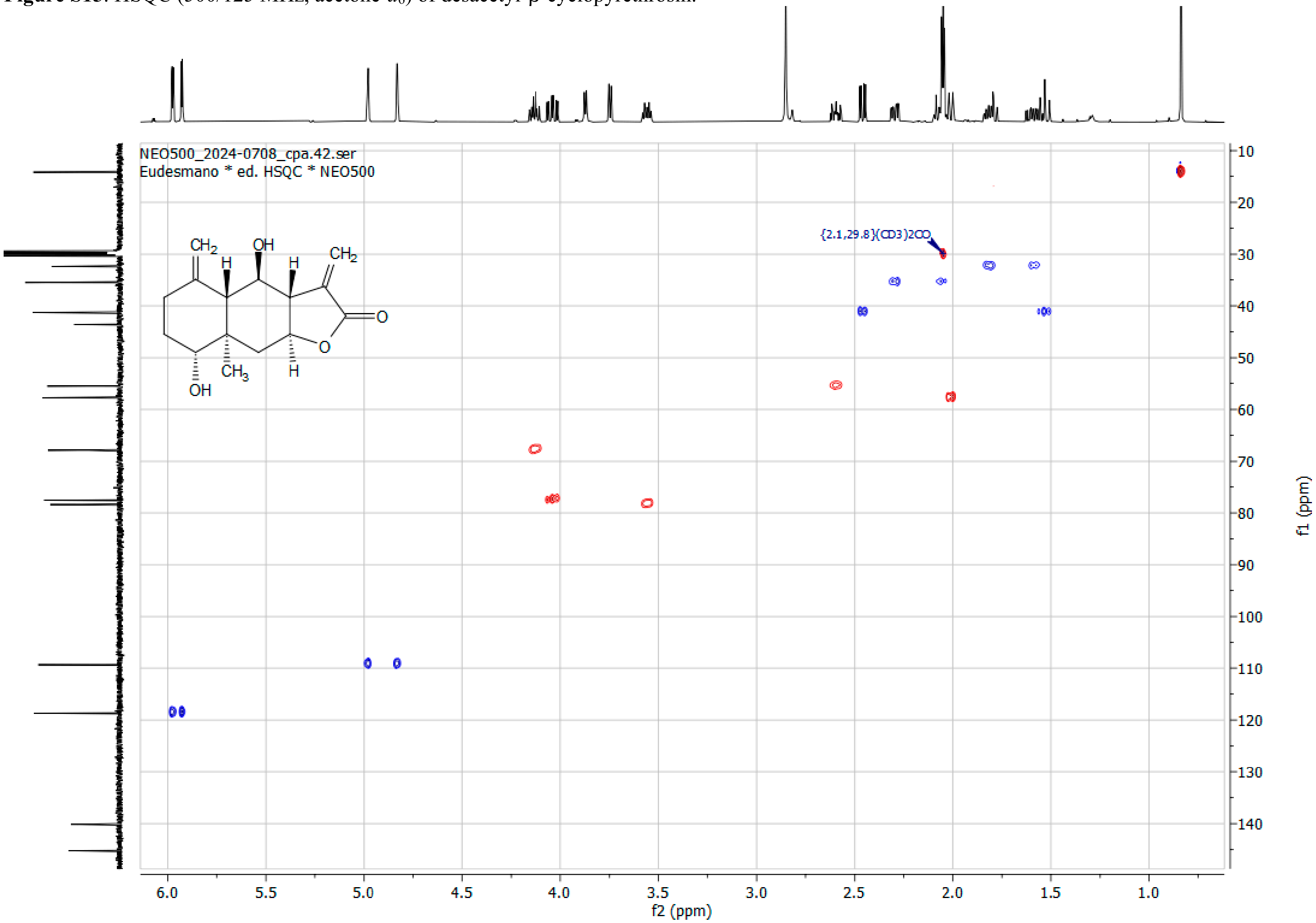

Figure S16. HMBC (500/125 MHz, acetone-d_6) of desacetyl-β-cyclopyrethrosin.

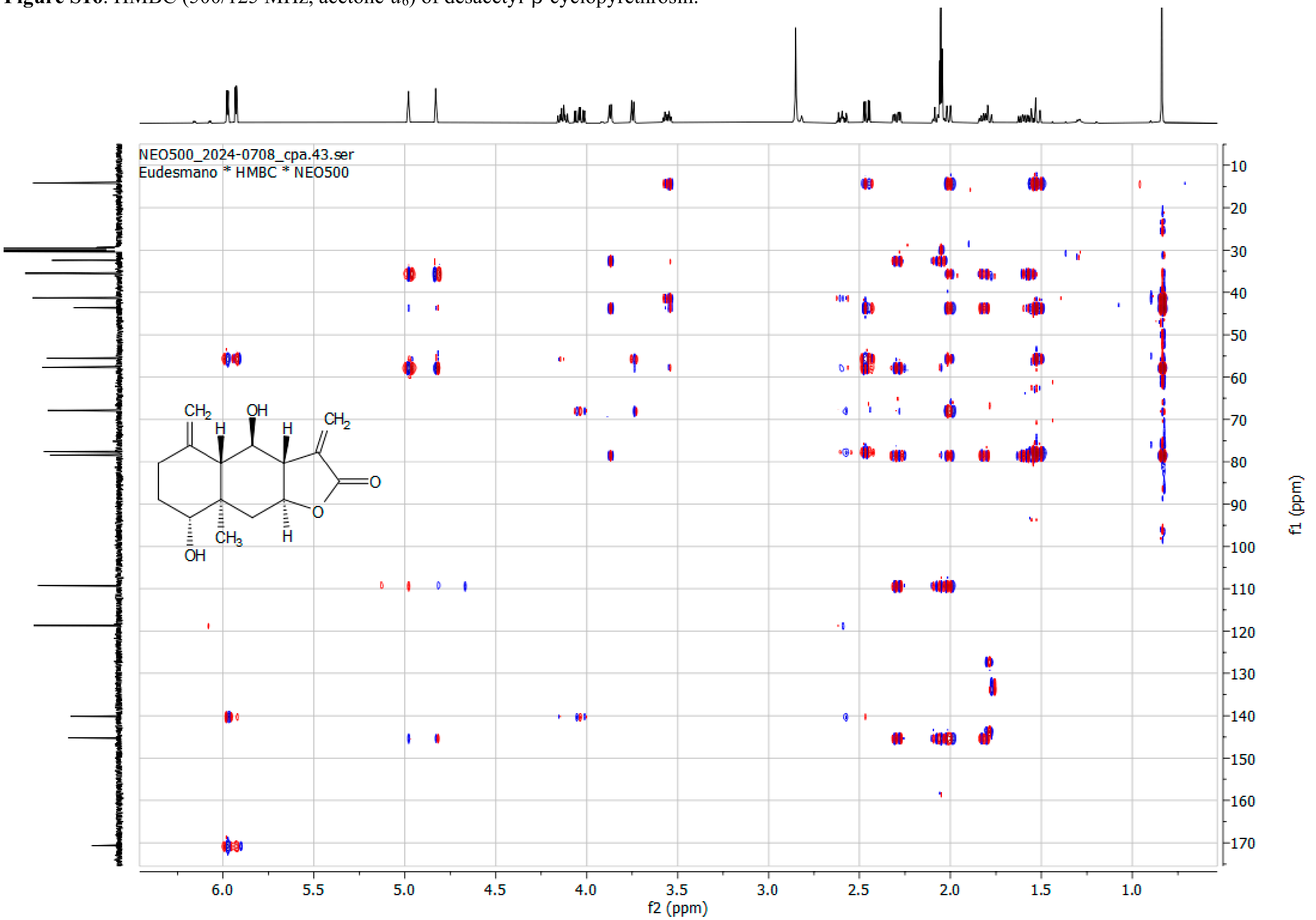

Figure S17. NOESY (500 MHz, acetone-d_6) of desacetyl-β-cyclopyrethrosin.

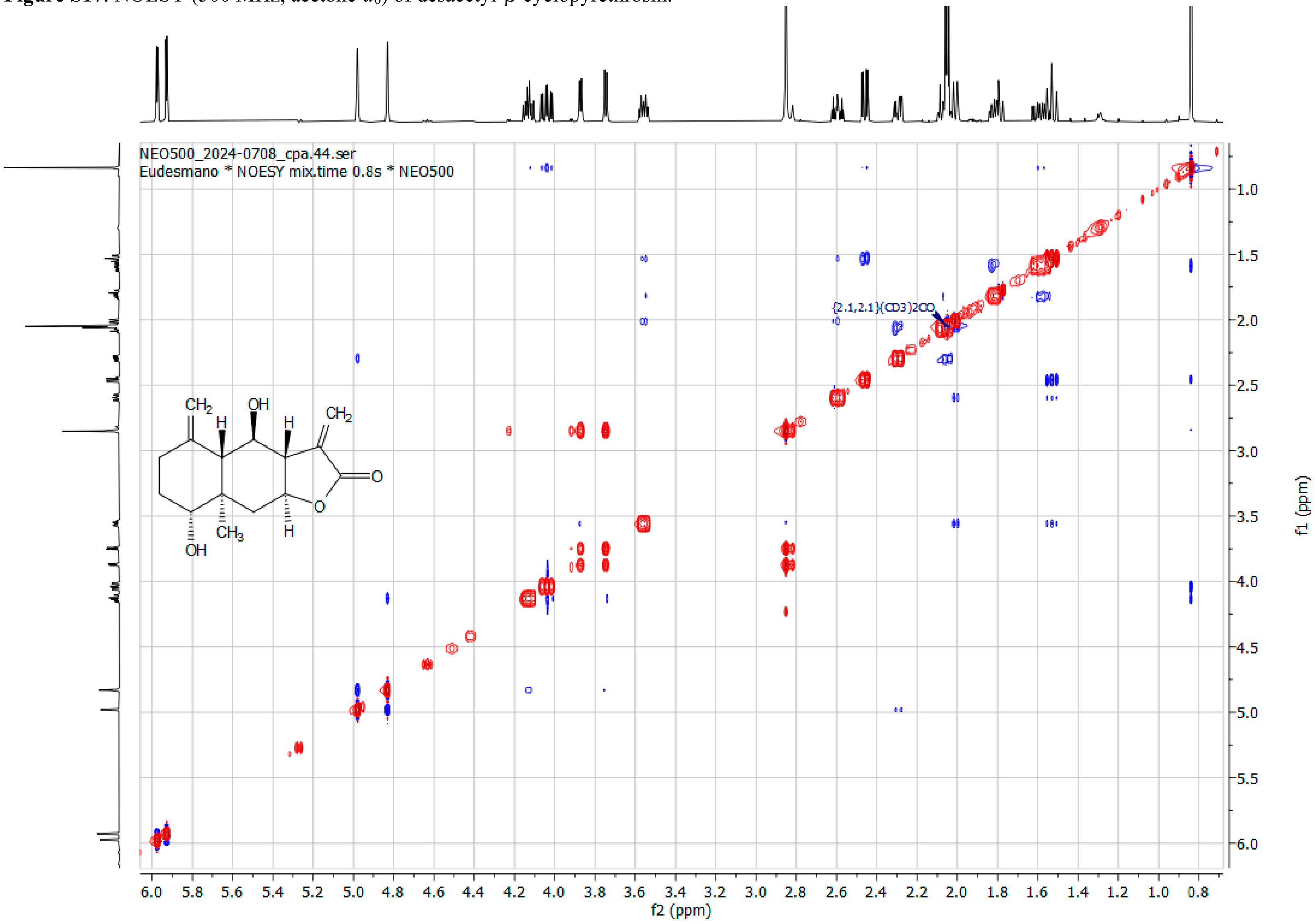

Figure S18. Original Western blot images of of IκBα, phospho-IκBα and α-Tubulin reported in Figure 6c

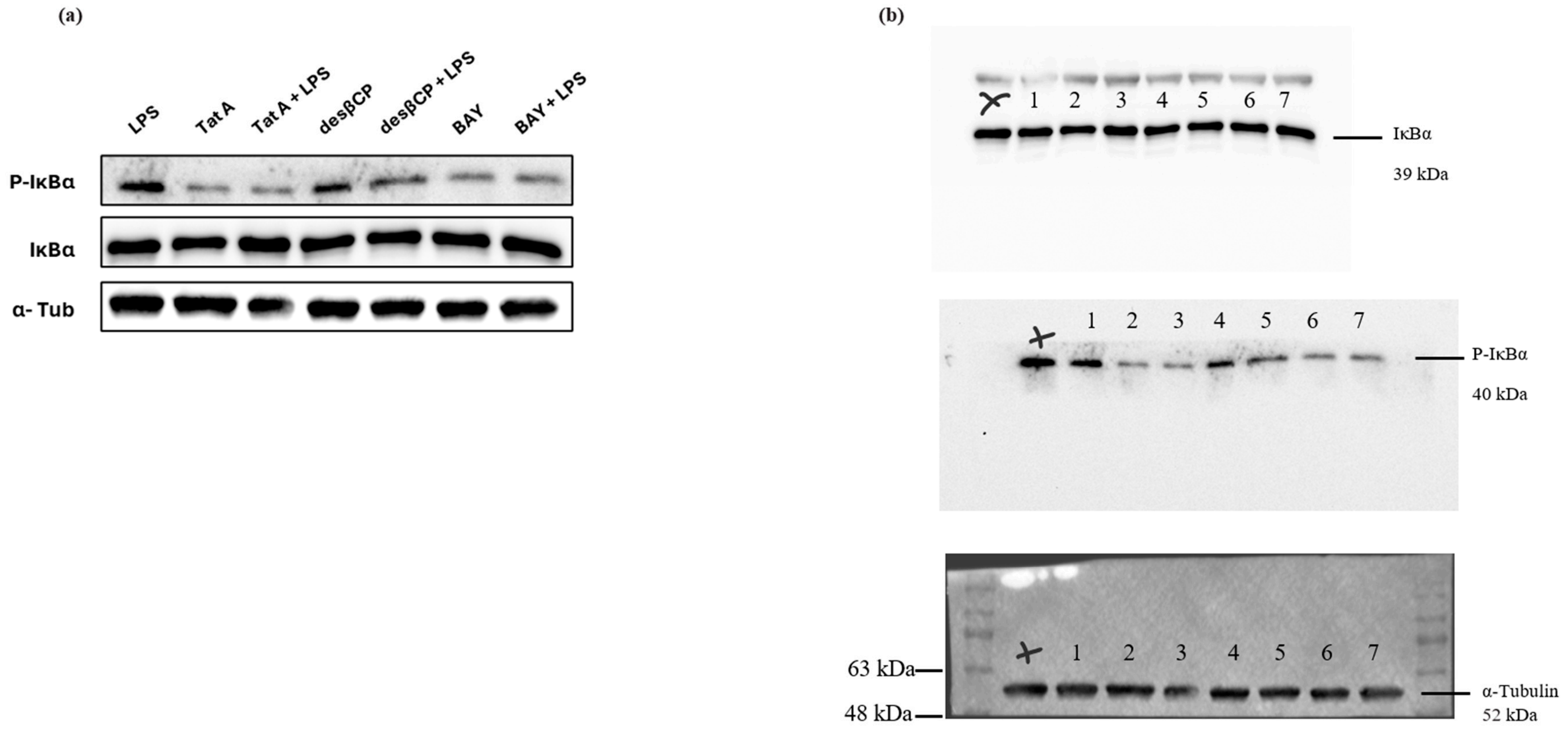

Figure S18. (a) Western blot images included in the manuscript (Figure 6c). **(b)** Related original Western blot images IκBα, phospho-IκBα and α-Tubulin reported as loading control.
